# Supplementary figures and images for: Dynamic large-scale connectivity of intrinsic cortical oscillations supports adaptive listening in challenging conditions
Source: PLoS Biol. 2021 Oct 11;19(10):e3001410. doi: 10.1371/journal.pbio.3001410 (PMC8530332; doi:10.1371/journal.pbio.3001410)

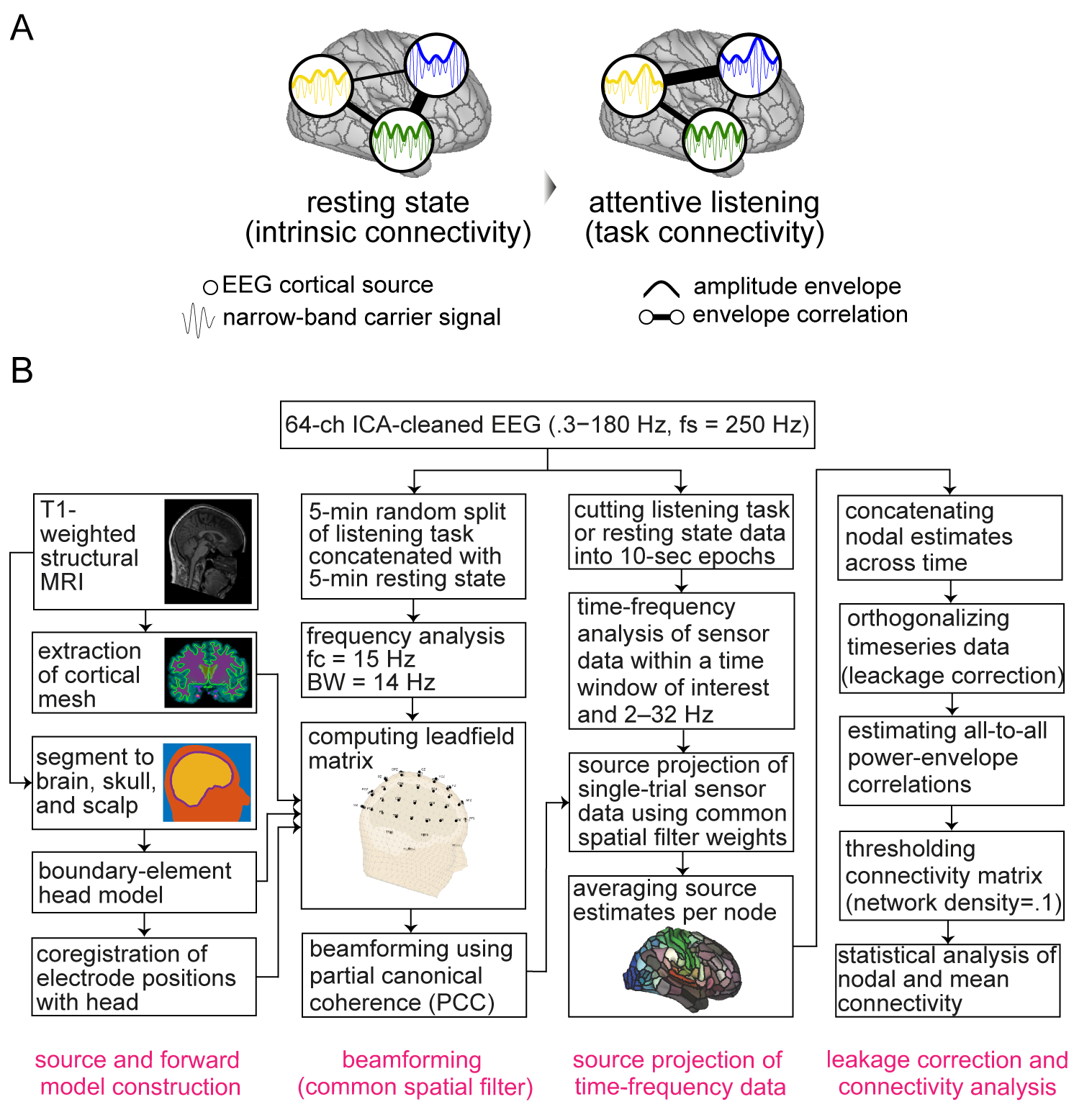

Supplement: S1 Fig — (A) Toy graphs illustrate connectivity between 3 exemplary EEG cortical sources as measured by power-envelope correlation. During attentive listening, intrinsic α/β neural oscillations reconfigure their putative baseline network (resting state) depending on the current task state to support listening behavior. These dynamics could manifest as change in connectivity strength (here depicted as edge thickness) or network segregation [5]. (B) EEG source analysis pipeline. Source reconstruction of EEG oscillatory responses and estimation of connectivity per individual (N = 154) were implemented in four steps: (1) construction of source geometry using MRI T1-derived cortical mesh and head boundary-element model (2) estimation of lead-field matrix and a spatial filter common across rest, task, and a broad frequency band (fc = 15 Hz; BW = 14 Hz) (3) source projection of time-frequency sensor data and averaging the source estimates per cortical parcel or node (4) pair-wise orthogonalization of complex-value time-frequency source estimates across all nodes to diminish spurious correlations due to volume conduction [25]. These steps at the end gave individual cortical connectivity maps per time-window of interest and frequency band. These analyses were performed using Fieldtrip toolbox for Matlab and HCP functional parcellation template [46]. BW, band-width; EEG, electroencephalography; fc, center frequency; fs, sampling frequency; HCP, Human Connectome Project; ICA, independent component analysis; MRI, magnetic resonance imaging; PCC, partial canonical coherence. (TIF) [file pbio.3001410.s001.tif]

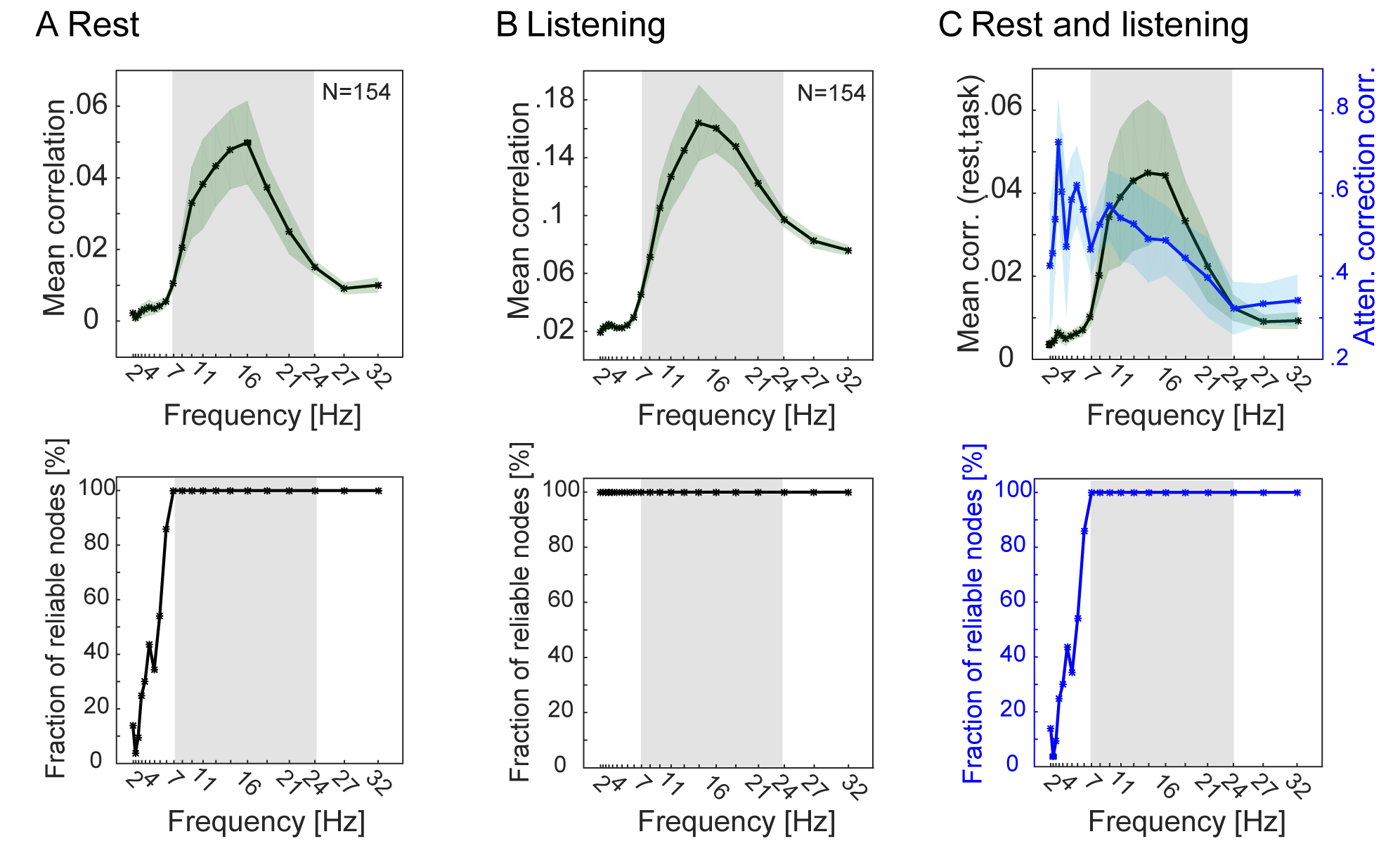

Supplement: S2 Fig — A serious but often neglected potential confounding factor in assessments of neuronal interactions is SNR [48,49]. This is an important issue in the present study as we compare connectivity of frequency-specific neural oscillations between resting state and listening task, each of which is potentially measured at different levels of SNR. Thus, we first investigated at which frequencies connectivity can be reliably measured under both rest and task conditions. As a measure of reliability, we used between-subject correlation of rest and task nodal connectivity values, i.e., columns of raw connectivity matrices. The reliability analysis was done in two steps. (A, B) First, per cortical node, correlations between nodal connectivity values were calculated across all pairs of N = 154 participants, separately for rest or task condition. This procedure per node gives one symmetric N × N correlation matrix for each rest or task condition. The upper-diagonal average of this matrix is mean between-subject correlation in nodal connectivity for each rest or task condition (within-condition reliability). The plots in (A) and (B) illustrate mean ± SEM of these between-subject correlations averaged across all 360 cortical nodes per frequency. (C) Next, the same analysis was done across rest and task. In this case, the result per node is one asymmetric N × N correlation matrix. The off-diagonal average of this matrix is mean between-subject correlation in nodal connectivity across rest and task (between-condition reliability). The black line graph in (C) illustrates mean ± SEM of these between-subject correlations averaged across all 360 cortical nodes per frequency. These correlations are underestimated in the presence of noise. Thus, they were submitted to Spearman’s correction for attenuation to account for differences in SNR between rest and task (blue line graph). Estimation of connectivity at a given frequency and node was considered reliable if the attenuation-corrected corre [file pbio.3001410.s002.tif]

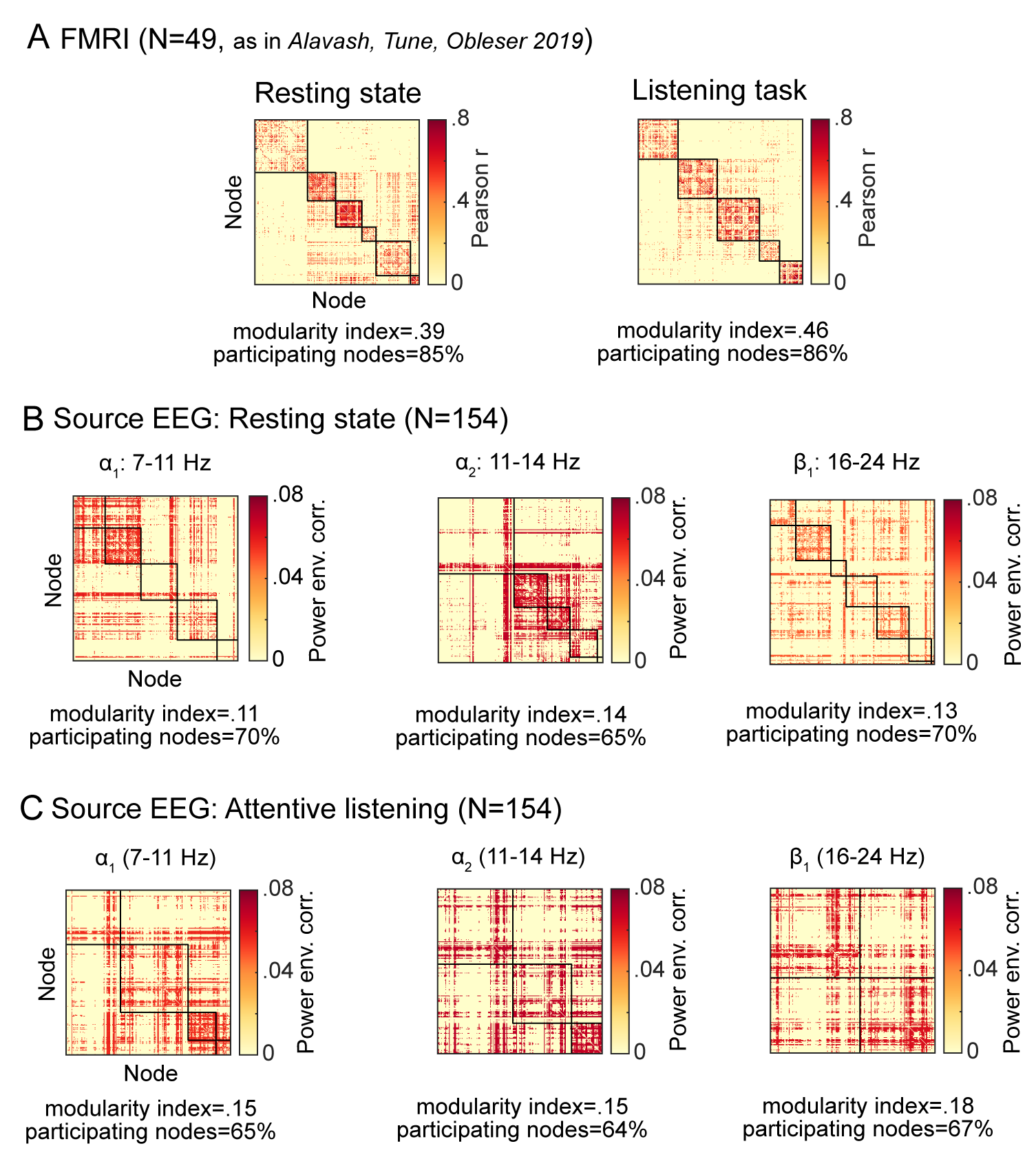

Supplement: S3 Fig — In each group-average connectivity matrix, diagonal squares represent functional modules. Off-diagonal correlations represent the strength of between-module connections. In (A), functional modules correspond to canonical resting state networks (left) and their makeup during the listening task (right; see [5]). In contrast, EEG source power-envelope correlations within alpha and low-beta bands did not exhibit a modular organization. Connectivity matrices are averaged across individuals and thresholded at 10% of network density. Modularity index (Q) quantifies the degree to which a network is clustered into densely intra-connected groups of nodes, which are sparsely inter-connected and is estimated based on Newman optimization algorithm [44,45] (the more modular the network, the closer the Q to 1). Percentage of participating nodes is calculated as the number of nodes having at least one connection in the network divided by the total number of nodes defined according to the cortical parcellation template [46]. The data underlying this figure can be found at https://osf.io/ge2cq/. EEG, electroencephalography; fMRI, functional magnetic resonance imaging. (TIF) [file pbio.3001410.s003.tif]

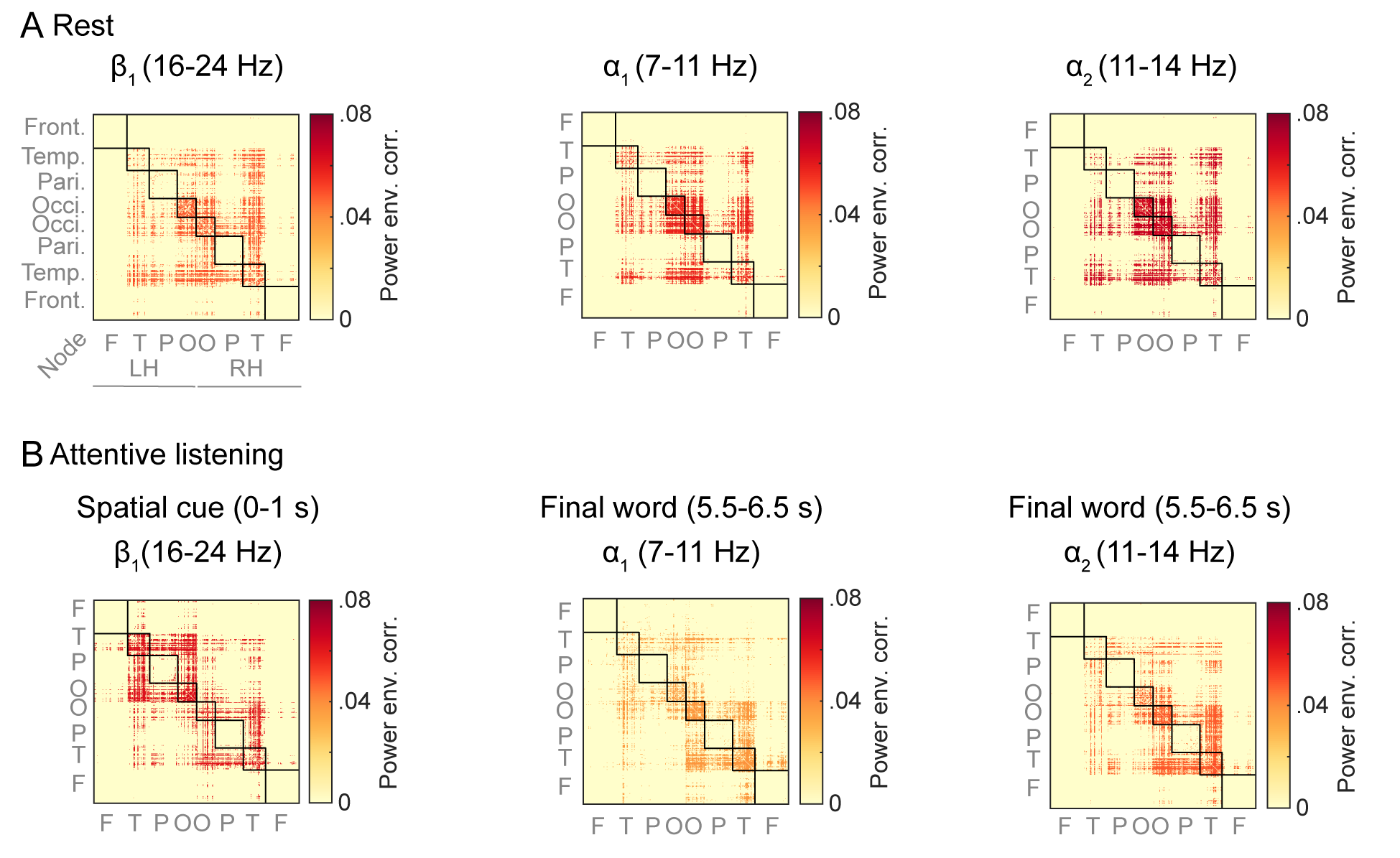

Supplement: S4 Fig — (A) For each frequency band, power-envelope correlations between EEG oscillatory sources were estimated using 4-min eyes-open resting state data. (B) Whole-brain connectivity during task time intervals most critical to listening behavior. Power-envelope correlations were estimated by concatenating 1-s windowed signals across all 240 trials (4-min data). Note the stronger beta-band connectivity during processing of spatial cue and weaker alpha-band connectivity during final word presentation in (B) relative to resting state (A). Connectivity maps were averaged across N = 154 individuals and thresholded at 10% of network density. Nodes correspond to cortical parcels as in [46] and are grouped according to their cortical lobes. The data underlying this figure can be found at https://osf.io/ge2cq/. EEG, electroencephalography; LH, left hemisphere; RH, right hemisphere. (TIF) [file pbio.3001410.s004.tif]

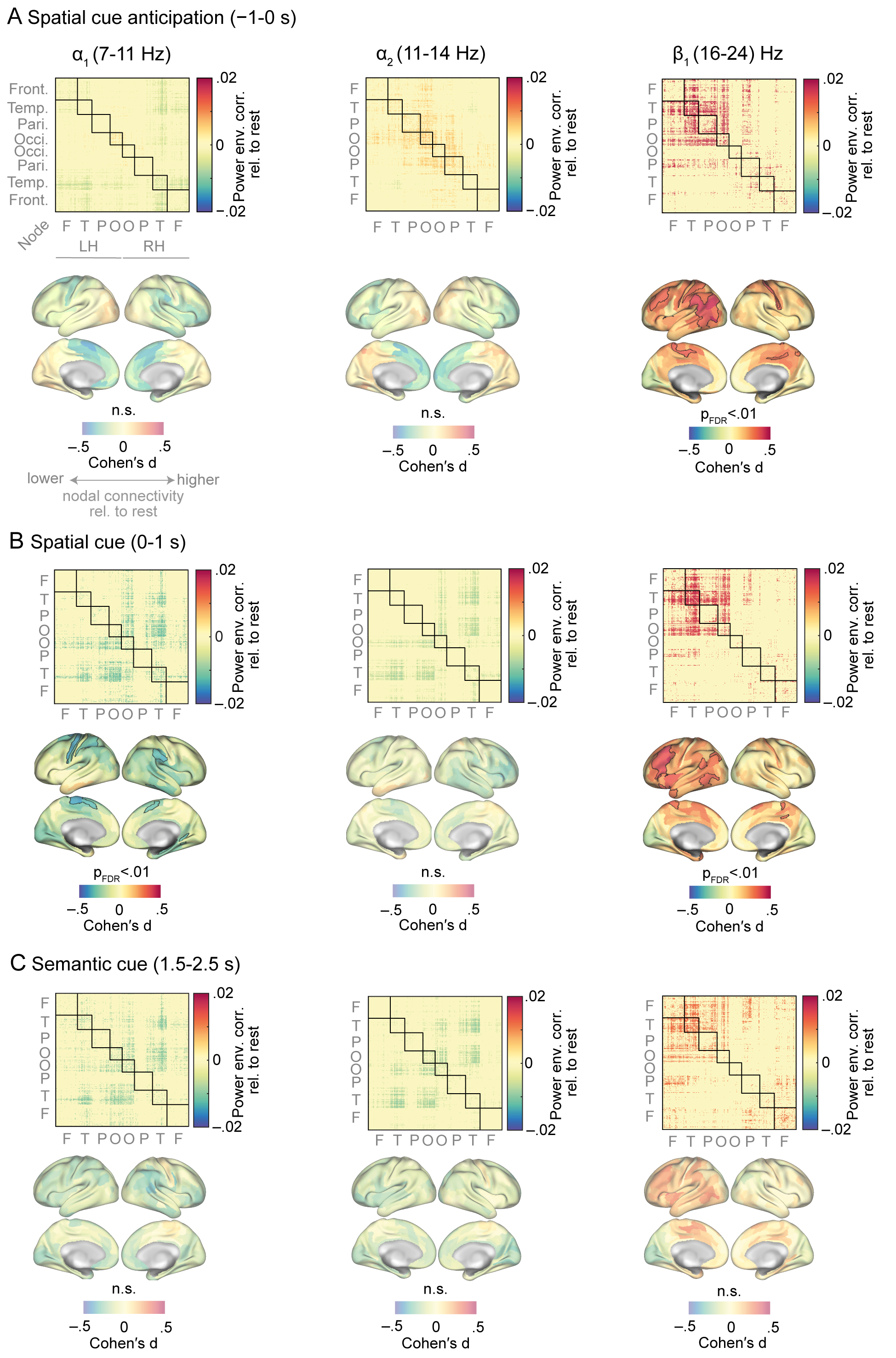

Supplement: S5 Fig — For each time interval (A-C) and frequency band, power-envelope correlations between EEG oscillatory sources were estimated by concatenating 1-s windowed signals across all 240 trials (4-min data) and compared with 4-min resting state connectivity at the same frequency band (i.e., task minus rest). In anticipation of and during the spatial cue presentation, β1 connectivity was significantly increased mainly across frontoparietal regions (A and B, third column; paired-sample permutation tests; significant nodes are outlined in black). Alpha-band connectivity was not significantly different than rest. Connectivity difference maps were averaged across N = 154 individuals and thresholded at 10% of network density. Nodes correspond to cortical parcels as in [46] and are grouped according to their cortical lobes. The data underlying this figure can be found at https://osf.io/ge2cq/. EEG, electroencephalography; LH, left hemisphere; RH, right hemisphere. (TIF) [file pbio.3001410.s005.tif]

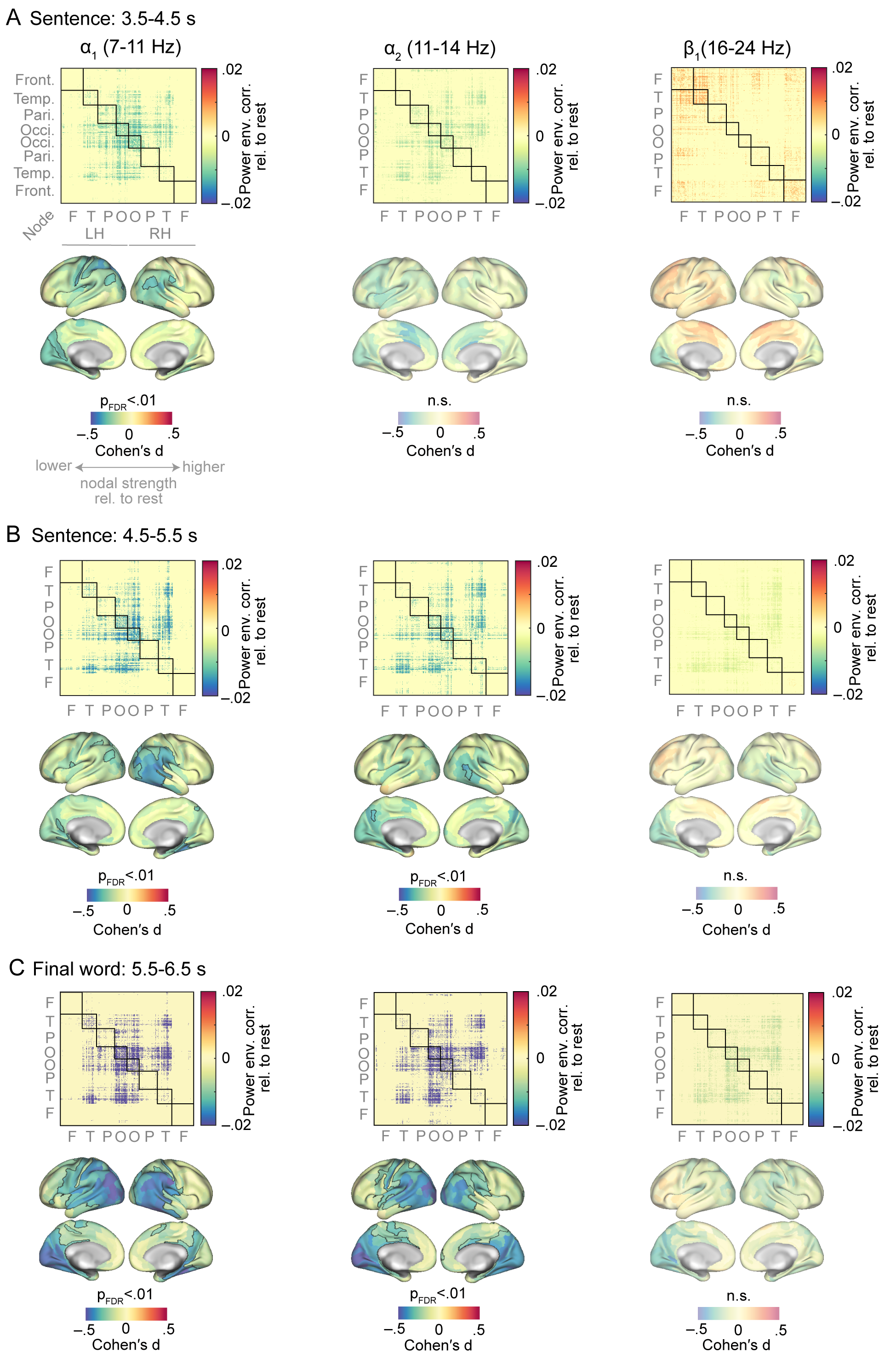

Supplement: S6 Fig — For each time interval of sentence presentation (A-C) and frequency band, power-envelope correlations between EEG oscillatory sources were estimated by concatenating 1-s windowed signals across all 240 trials (4-min data) and compared with 4-min resting state connectivity at the same frequency band (i.e., task minus rest). Toward the end of sentence and particularly during final word presentation, alpha-band connectivity was significantly decreased across posterior cortical regions (A-C, first column; paired-sample permutation tests; significant nodes are outlined in black). Beta-band connectivity was not significantly different than rest during sentence presentation. Connectivity difference maps were averaged across N = 154 individuals and thresholded at 10% of network density. Nodes correspond to cortical parcels as in [46] and are grouped according to their cortical lobes. The data underlying this figure can be found at https://osf.io/ge2cq/. EEG, electroencephalography; LH, left hemisphere; RH: right hemisphere. (TIF) [file pbio.3001410.s006.tif]

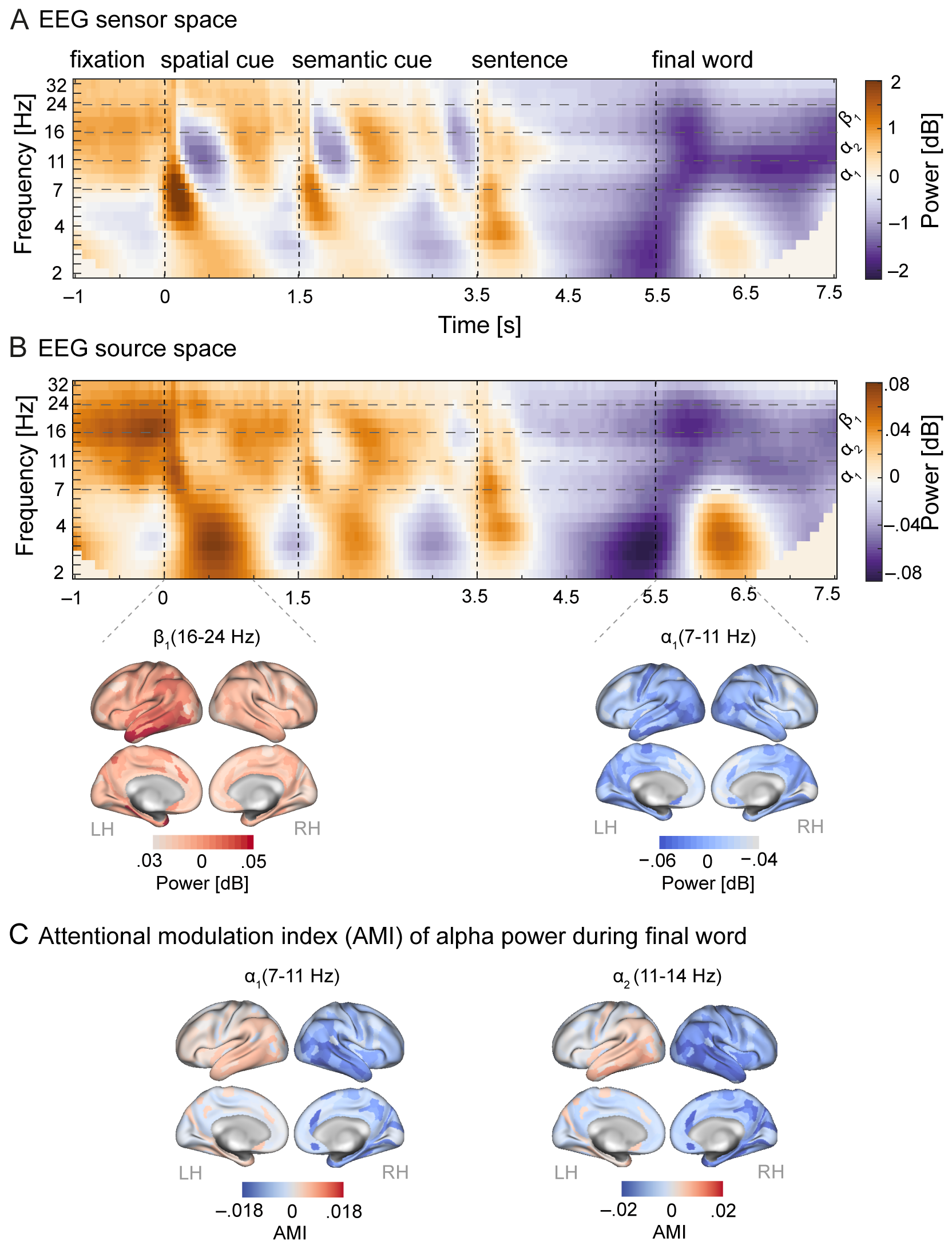

Supplement: S7 Fig — (A) Time-frequency representation in EEG sensor space. Power estimates were averaged across all sensors, trials, and N = 154 participants. (B) The same as in (A) but based on power of EEG source estimates and averaged across all cortical nodes. Power changes are calculated as dB change relative to the whole-trial baseline interval (−1–7.5 s). (C) Attentional modulation of alpha source power during selective attention to final word. The modulation index is calculated as AMI = (α-powerattendL − α-powerattendR) / (α-powerattendL + α-powerattendR). The data underlying this figure can be found at https://osf.io/ge2cq/. AMI, attentional modulation index; EEG, electroencephalography; LH, left hemisphere; RH, right hemisphere. (TIF) [file pbio.3001410.s007.tif]

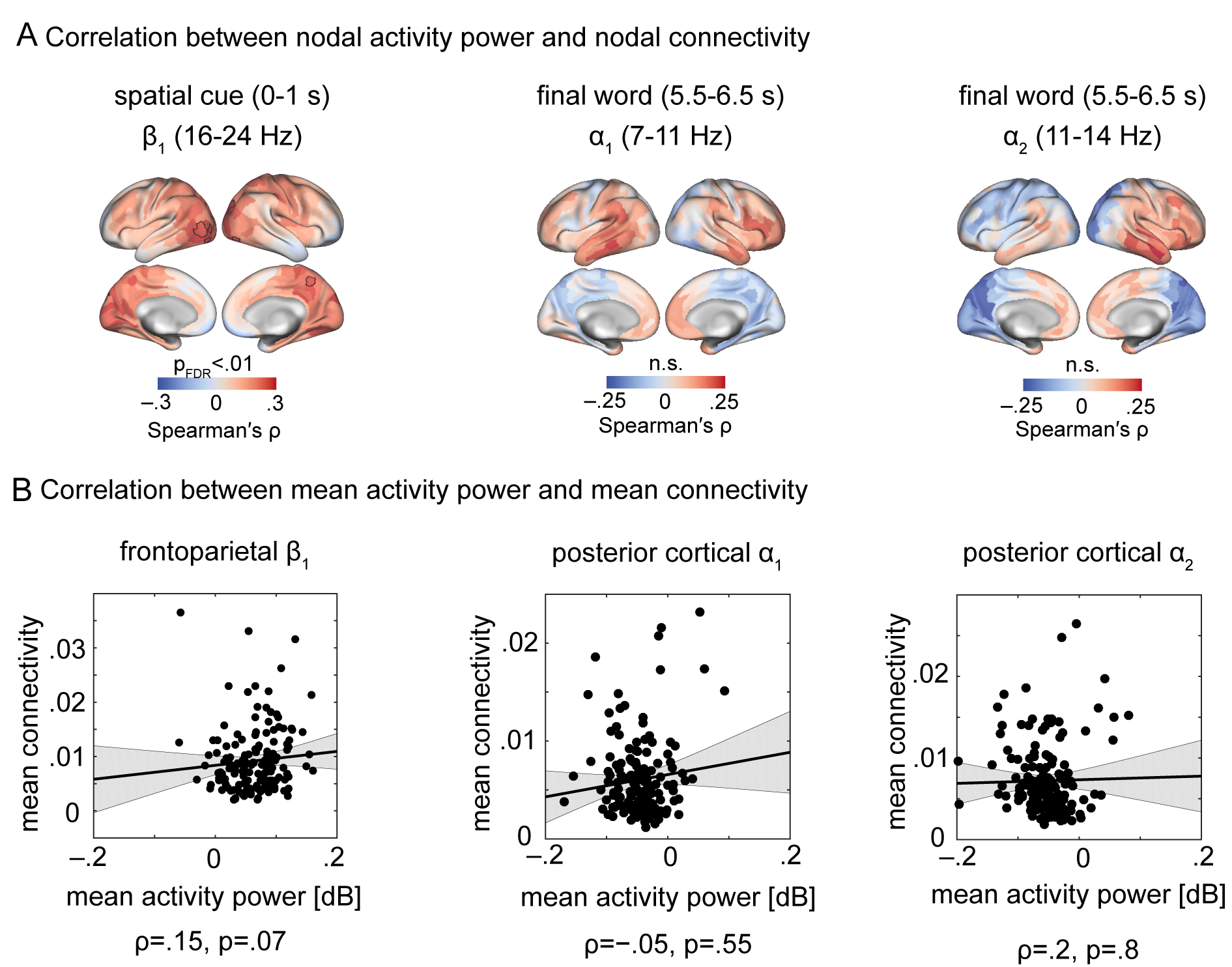

Supplement: S8 Fig — (A) For each frequency band and time interval, correlation between nodal connectivity and mean nodal power (dB) was tested across all N = 154 participants and per cortical node. (B) The same analysis as in (A) but using measures averaged across frontoparietal or posterior cortical nodes involved in beta-band hyperconnectivity or alpha-band hypoconnectivity, respectively. The data underlying this figure can be found at https://osf.io/ge2cq/. n.s., not significant. (TIF) [file pbio.3001410.s008.tif]

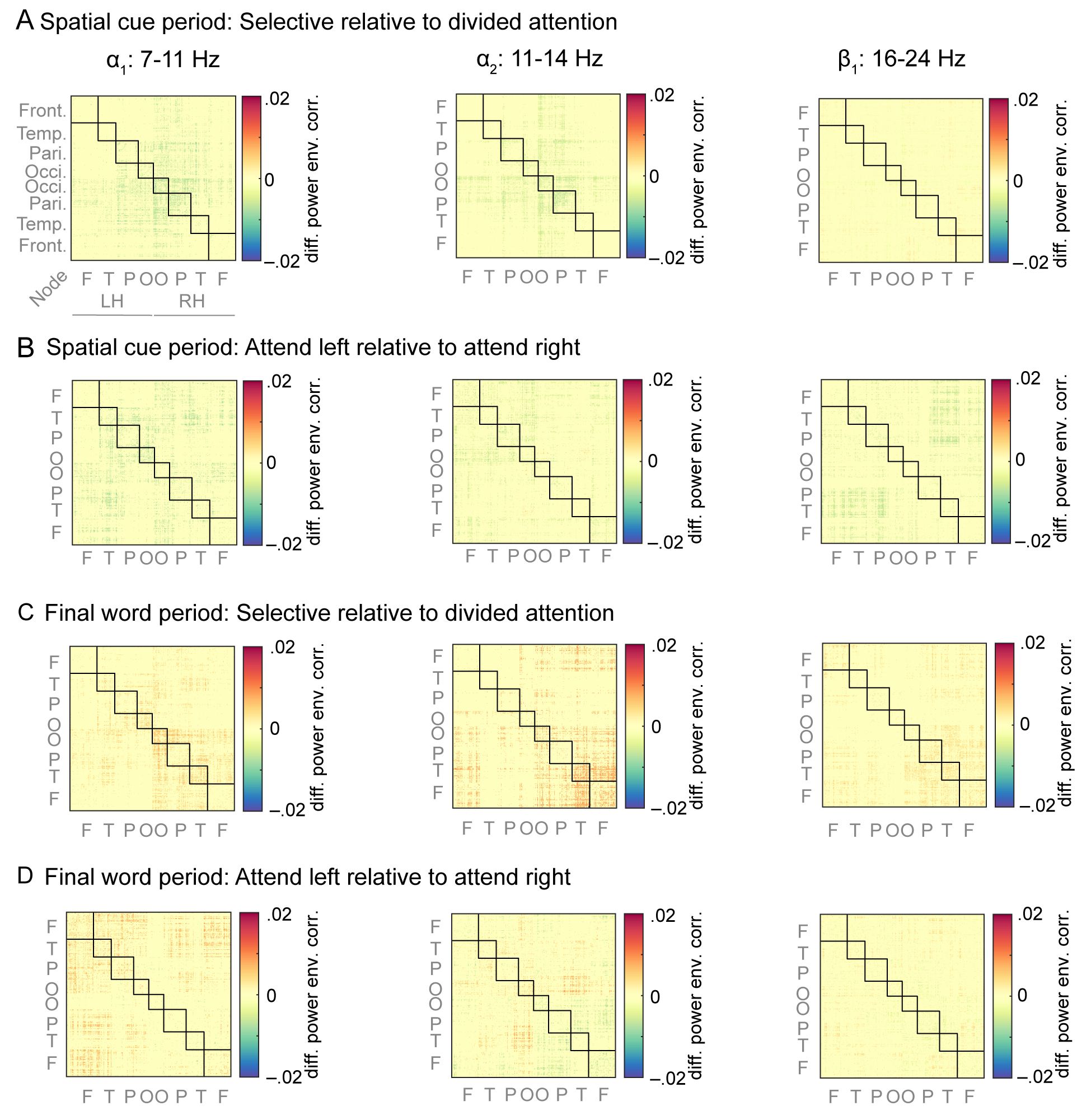

Supplement: S9 Fig — We investigated whether alpha- or beta-band connectivity showed a similar hemispheric lateralization in response to the spatial cue as in alpha power. For each spatial-cue (A, B) or final-word (C, D) time interval and frequency band, power-envelope correlations between EEG oscillatory sources were estimated by concatenating 1-s windowed signals across trials per spatial-cue condition: selective attention (i.e., attend left or right), divided attention, attend left, or attend right. The results were then compared by first calculating connectivity difference maps per participant (e.g., selective minus divided) and then averaging the difference maps across N = 154 individuals. If connectivity is confounded by activity level, these maps should reveal differences in power-envelope correlations between conditions. In contrast, power-envelope correlations were not influenced by attentional-cue conditions in neither of the trial intervals nor frequency bands. None of the connectivity contrasts revealed significant differences in nodal connectivity when tested across all cortical nodes using paired-sample permutation tests. Nodes correspond to cortical parcels as in [46] and are grouped according to their cortical lobes. The data underlying this figure can be found at https://osf.io/ge2cq/. EEG, electroencephalography; LH, left hemisphere; RH, right hemisphere. (TIF) [file pbio.3001410.s009.tif]

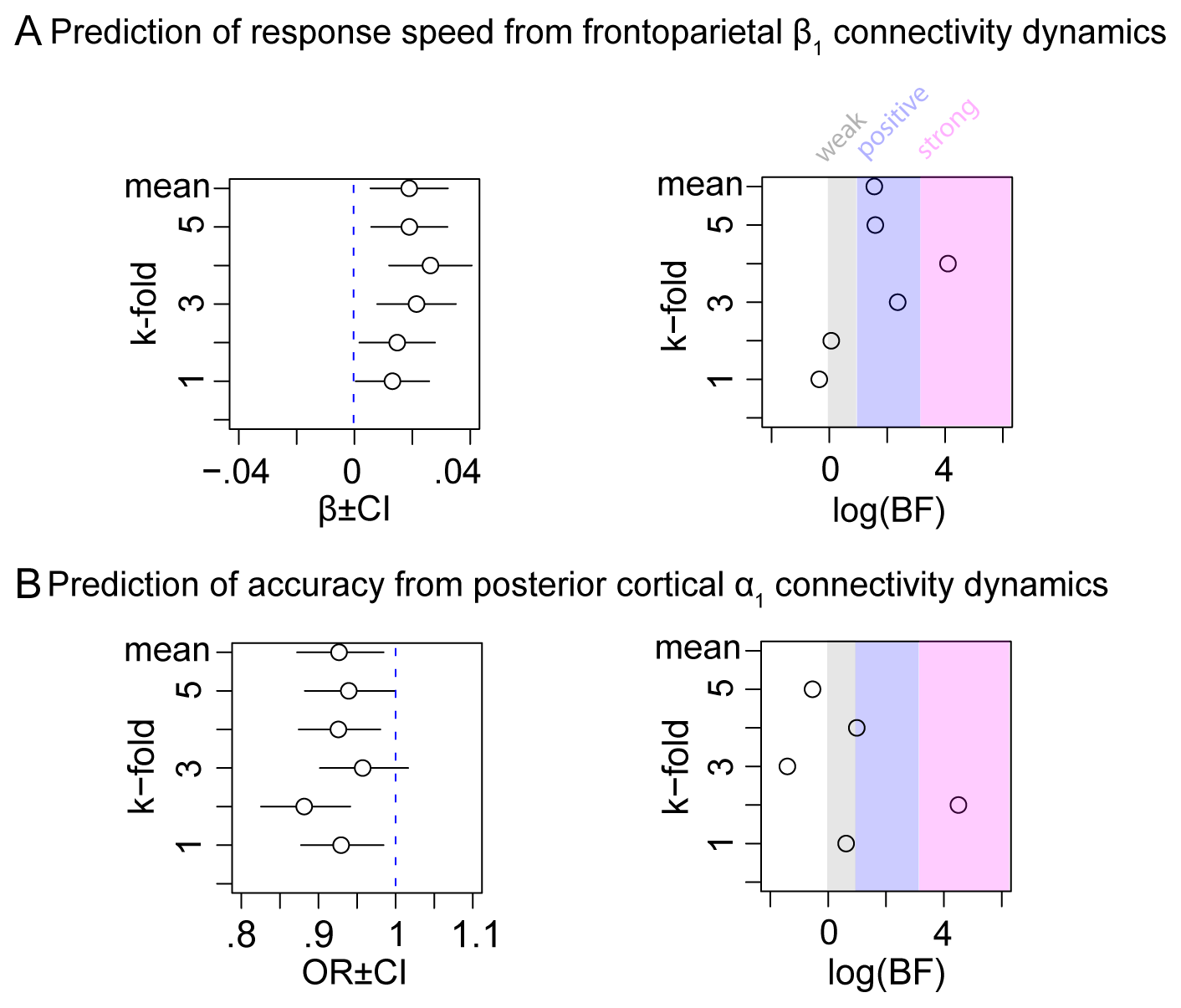

Supplement: S10 Fig — The reliability and robustness of the brain-behavior interaction effects (main text, Fig 6) were investigated by randomly splitting the data into k = 5 nonoverlapping folds. The strength and uncertainty (i.e., confidence interval) of the model’s parameter estimate was then examined when each fold of data was used. Since this analysis does not take the number of observations and model complexity into account, it could be that the model has a relatively high goodness of fit as a too complex model is fitted to the data. We thus examined the relative strength of evidence (rather than statistical significance) in support of the alternative hypothesis (i.e., model with the interaction term) as compared to the null hypothesis (i.e., model without the interaction term). This was done by calculating the BIC approximation of the BF, i.e., exp([BIC(H0)−BIC(H1)]/2). In this calculation, BIC imposes penalty on each model log-likelihood based on both number of observations and parameters. Following Harrold Jeffery’s scale for interpretation of BF, 0 < log-BF < 1 suggests weak evidence for H1, 1 < log-BF < 3 is positive, and 3 < log-BF < 5 is strong [104]. β: Slope parameter estimates from linear mixed-effects model. OR: odds ratio parameter estimates from generalized linear mixed-effects models. The data underlying this figure can be found at https://osf.io/ge2cq/. BF, Bayes factor; BIC, Bayesian information criterion; OR, odds ratio. (TIF) [file pbio.3001410.s010.tif]

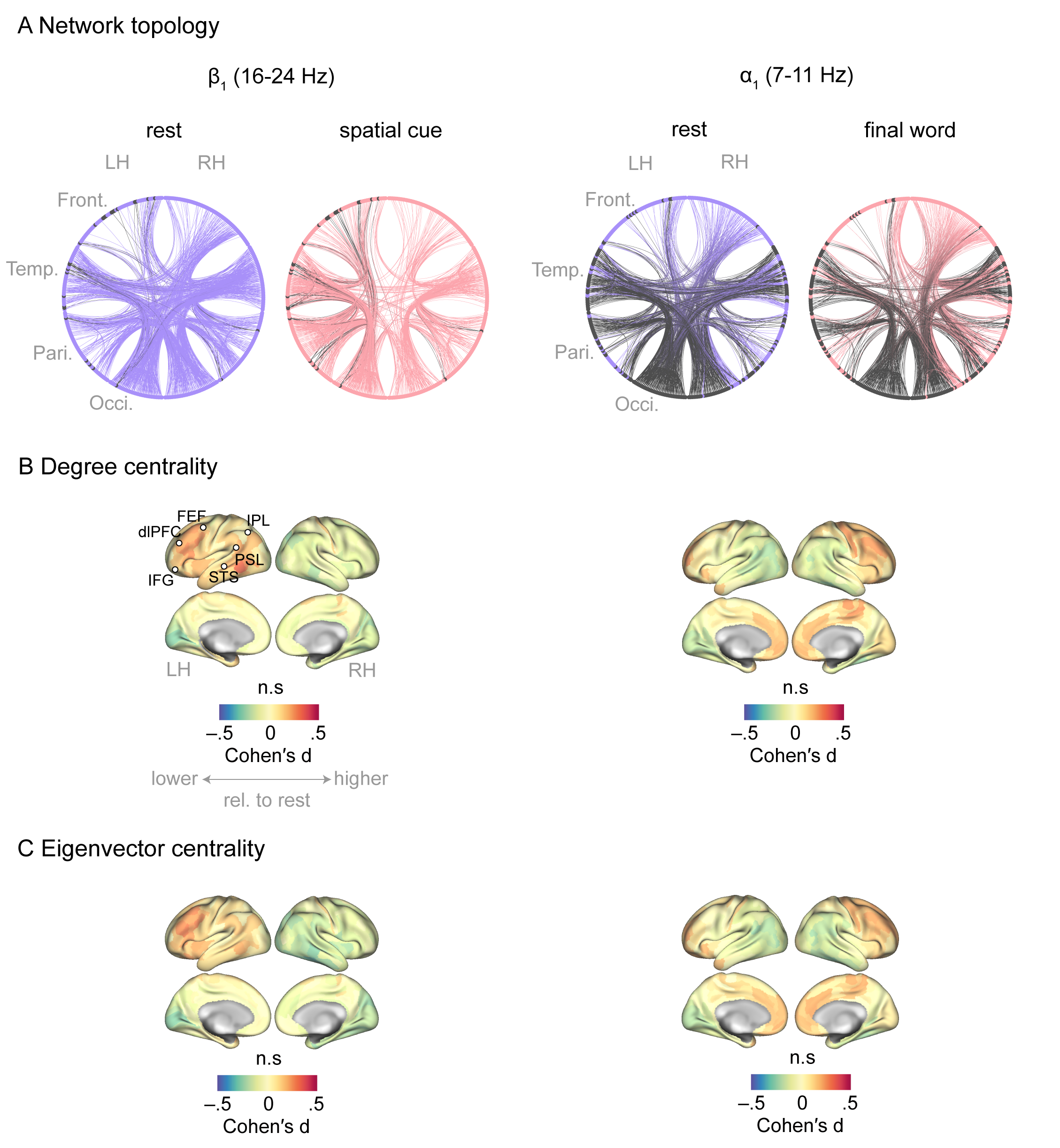

Supplement: S11 Fig — (A) Network topology per frequency band and condition. Each connectogram corresponds to its connectivity matrix counterpart and is obtained by setting the connection weights to 1 for correlations higher than 95th percentile, and to 0 otherwise (i.e., a sparse binary graph thresholded at 5% of density). Nodes correspond to cortical parcels as in [46] and are grouped according to their cortical lobes. The frontoparietal and posterior cortical nodes showing significant modulation of β1 and α1 connectivity strength, respectively (main text, Fig 5), are depicted in black. (B) Comparison of nodal degree centrality between each task interval and rest using permutation tests. Degree centrality is defined as each node’s number of connections (also referred to as node’s degree [44]). This measure quantifies relative importance of nodes: Nodes having large number of connections, hence high degree centrality, can be considered as central coordinators in the functional network. In contrast to nodal connectivity strength, nodal degree was not significantly modulated when tested across all cortical nodes (brain surfaces; significance level: pFDR < 0.01). (C) Comparison of eigenvector centrality. This metric is more specific than degree centrality as it also takes the relative importance of a node’s neighbors into account. Eigenvector centrality of node i is equivalent to the ith element in the eigenvector corresponding to the largest eigenvalue of the adjacency matrix. Nodes having high eigenvector centrality are high-degree nodes whose neighbors also have relatively large number of connections. Eigenvector centrality was not significantly modulated when tested across all cortical nodes. The data underlying this figure can be found at https://osf.io/ge2cq/. dlPFC, dorsolateral prefrontal cortex; FEF, frontal eye field; IFG, inferior frontal gyrus; IPL, inferior parietal lobule; LH, left hemisphere; n.s., not significant; PSL, perisylvian language area; RH, right hemisphere; STS, s [file pbio.3001410.s011.tif]

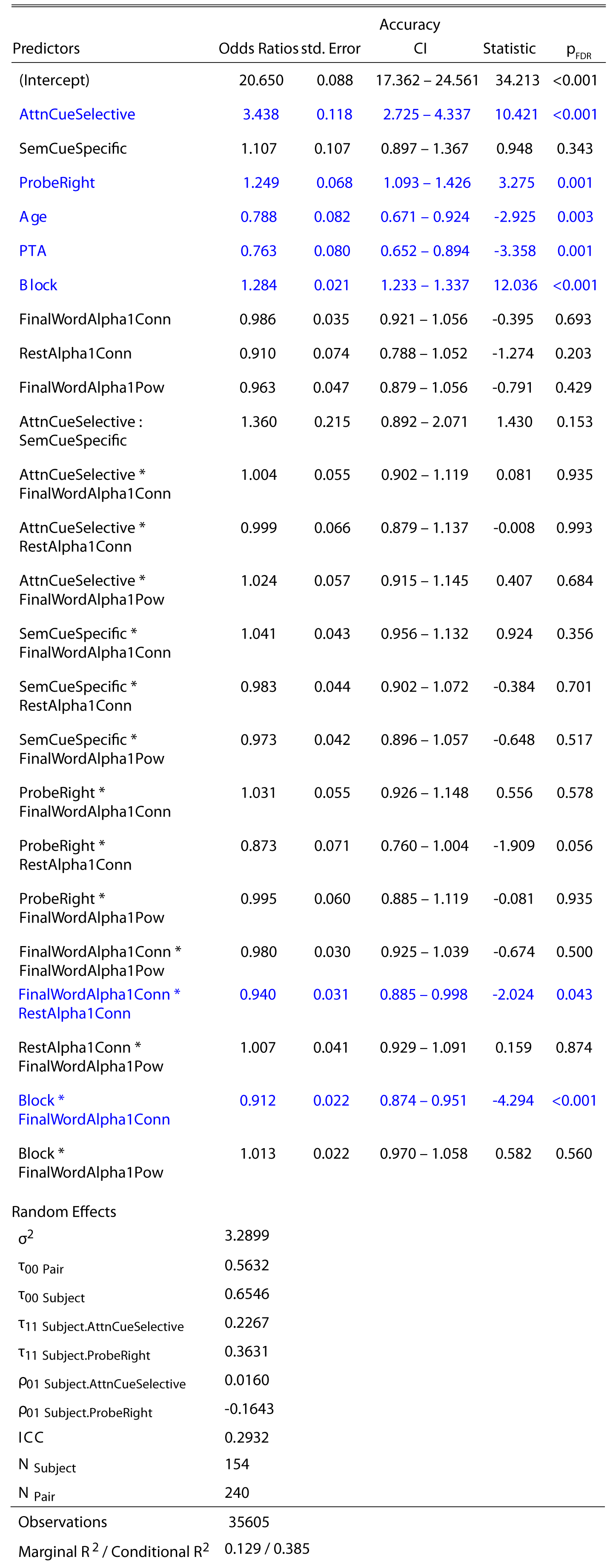

Supplement: S1 Table — In this model, brain regressors were based on the data during final word period and within α1 band. Significant effects after FDR-correction for multiple comparisons across model terms are shown in blue. The main effects of and interactions between listening cues are visualized in Fig 2 (main text). The interaction between rest and task connectivity is visualized in Fig 6. OR: odds ratio; σ2: within-group variance; τ00: between-group variance; ρ01: random-slope-intercept-correlation. FDR, false discovery rate; OR, odds ratio. (TIF) [file pbio.3001410.s012.tif]

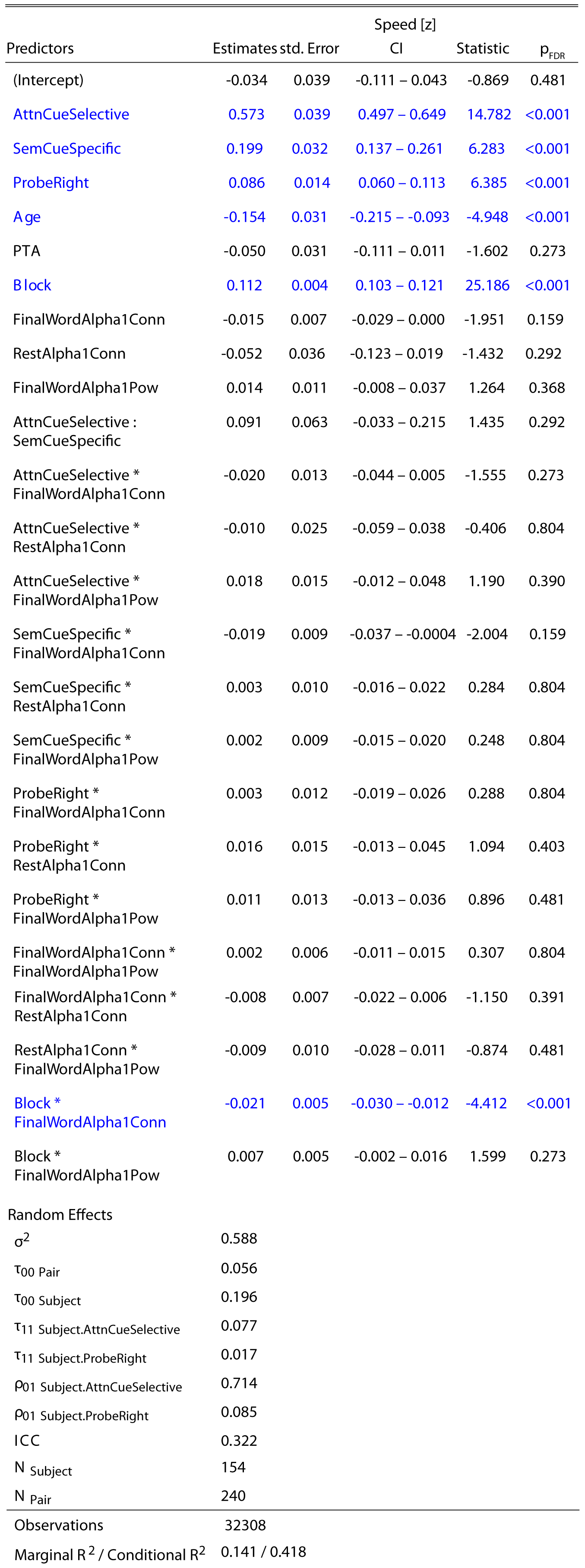

Supplement: S2 Table — In this model, brain regressors were based on the data during final word period and within α1 band. Significant effects after FDR-correction for multiple comparisons across model terms are shown in blue. The main effects of and interactions between listening cues are visualized in Fig 2 (main text). β: slope parameter estimate; σ2: within-group variance; τ00: between-group variance; ρ01: random-slope-intercept-correlation. FDR, false discovery rate. (TIF) [file pbio.3001410.s013.tif]

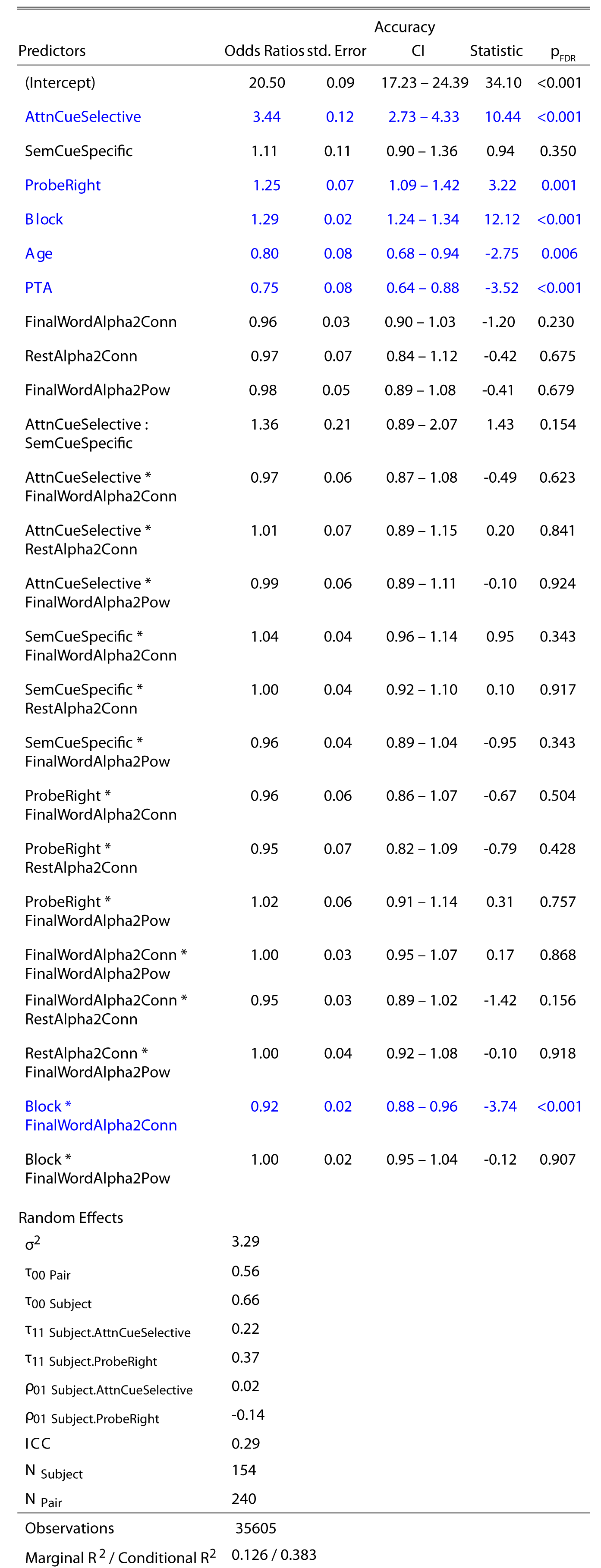

Supplement: S3 Table — In this model, brain regressors were based on the data during final word period and within α2 band. Significant effects after FDR-correction for multiple comparisons across model terms are shown in blue. OR: odds ratio; σ2: within-group variance; τ00: between-group variance; ρ01: random-slope-intercept-correlation. FDR, false discovery rate; OR, odds ratio. (TIF) [file pbio.3001410.s014.tif]

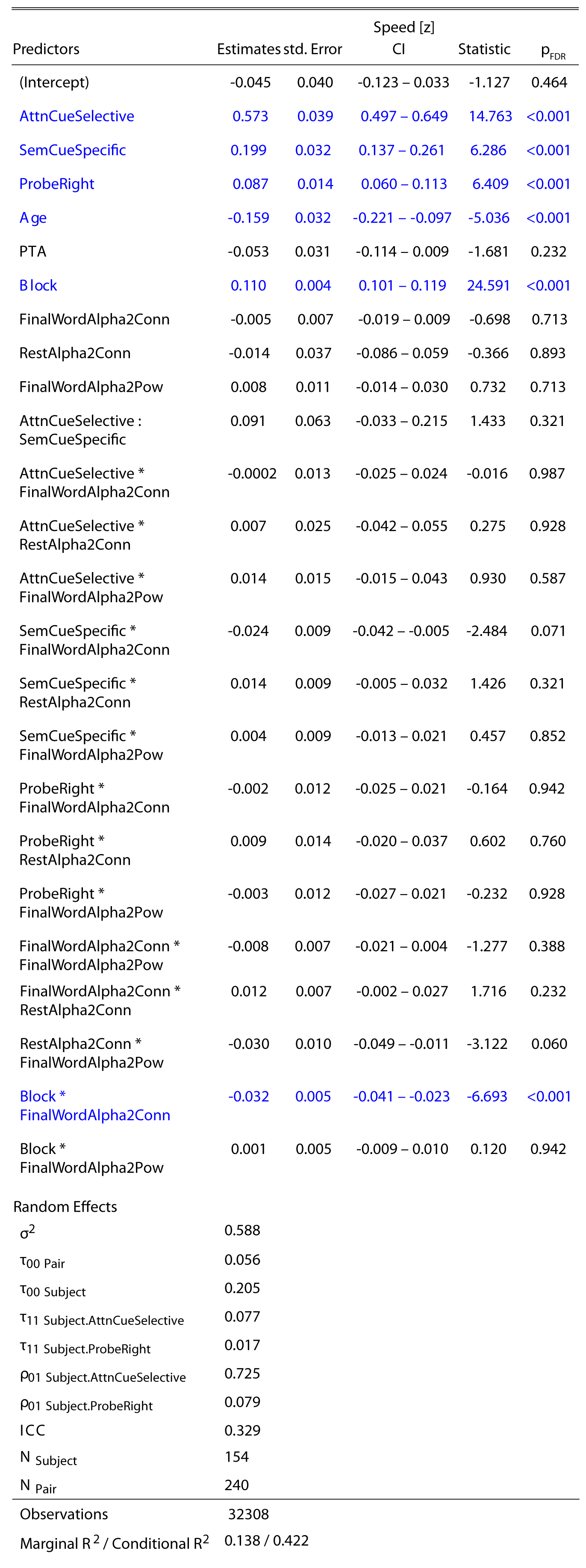

Supplement: S4 Table — In this model, brain regressors were based on the data during final word period and within α2 band. Significant effects after FDR-correction for multiple comparisons across model terms are shown in blue. β: slope parameter estimate; σ2: within-group variance; τ00: between-group variance; ρ01: random-slope-intercept-correlation. FDR, false discovery rate. (TIF) [file pbio.3001410.s015.tif]

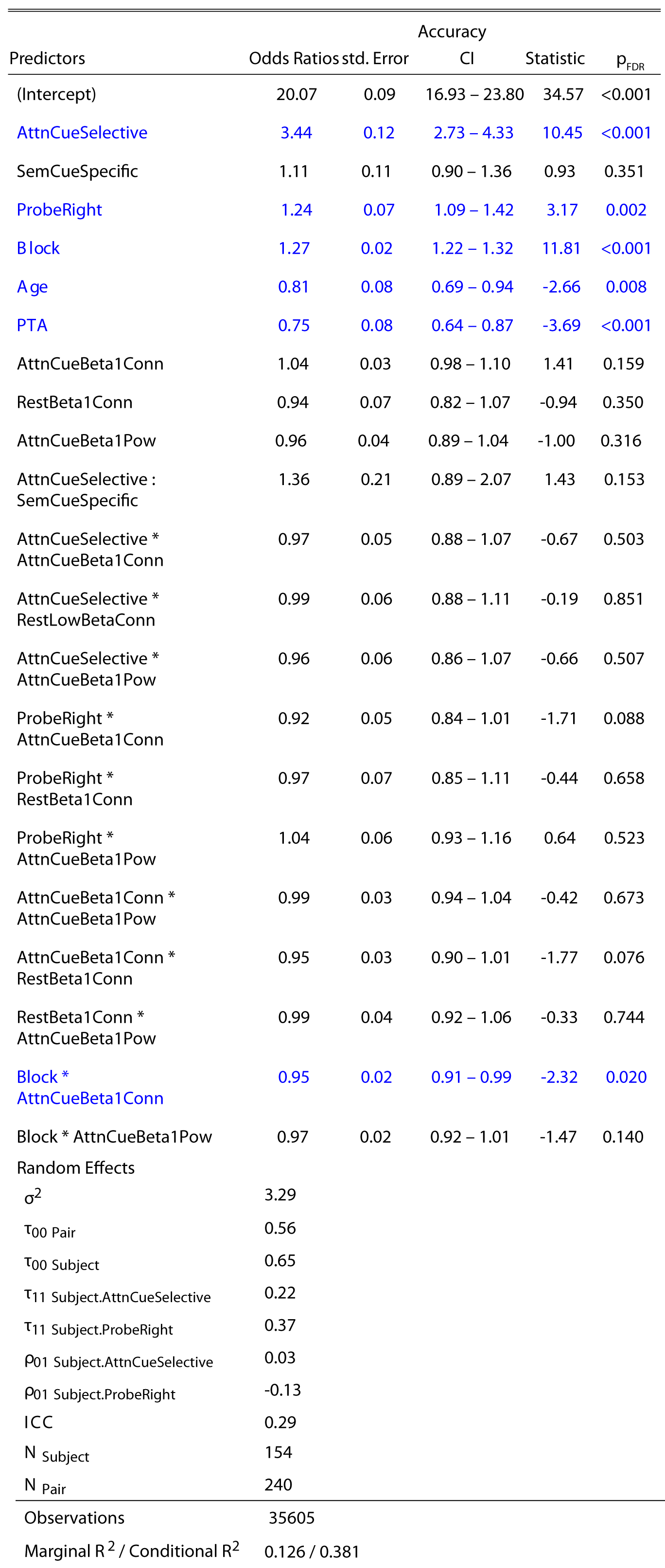

Supplement: S5 Table — In this model, brain regressors were based on the data during spatial cue and within β1 band. Significant effects after FDR-correction for multiple comparisons across model terms are shown in blue. OR: odds ratio; σ2: within-group variance; τ00: between-group variance; ρ01: random-slope-intercept-correlation. FDR, false discovery rate; OR, odds ratio. (TIF) [file pbio.3001410.s016.tif]

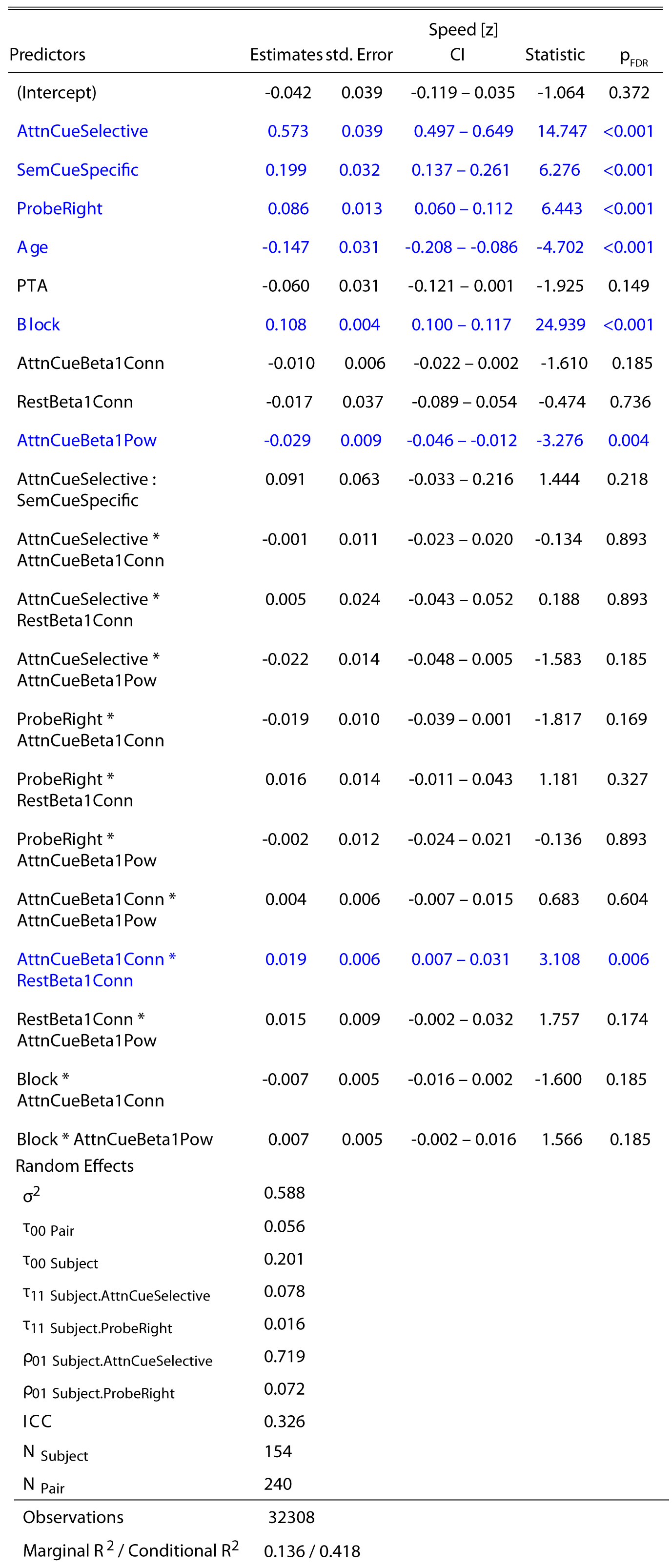

Supplement: S6 Table — In this model, brain regressors were based on the data during spatial cue period and within β1 band. Significant effects after FDR-correction for multiple comparisons across model terms are shown in blue. The main effects of and interactions between listening cues are visualized in Fig 2 (main text). The interaction between rest and task connectivity is visualized in Fig 6. β: slope parameter estimate; σ2: within-group variance; τ00: between-group variance; ρ01: random-slope-intercept-correlation. FDR, false discovery rate. (TIF) [file pbio.3001410.s017.tif]
