# Supplementary material for: Dynamic large-scale connectivity of intrinsic cortical oscillations supports adaptive listening in challenging conditions
Source: PLoS Biol. 2021 Oct 11;19(10):e3001410. doi: 10.1371/journal.pbio.3001410 (PMC8530332; doi:10.1371/journal.pbio.3001410)
Supplement: Attachment — Submitted filename: Response_to_reviews_R1.docx [file pbio.3001410.s018.docx]

Response letter to the reviewers’ comments on “Large-scale cortical connectivity dynamics of intrinsic neural oscillations support adaptive listening behavior” submitted to *PLOS Biology* in March 2021.

Manuscript No.: PBIOLOGY-D-21-00518R1

Authors: Mohsen Alavash*, Sarah Tune, Jonas Obleser*

*Corresponding author: Department of Psychology, University of Lübeck, 23562 Lübeck, Germany

Revised manuscript submitted to journal in Juli 2021.

**Reviewer 1**

“Alavash et al. re-analyzed this large dataset to address the question how EEG (power and) connectivity in alpha and beta frequency bands affect speech comprehension in a dichotic listening task. The study used an age-varying large sample of N=154 / N=127 after exclusion (I think). The authors found that in individuals with low frontoparietal beta connectivity during rest, there was a link between response speed and this connectivity during spatial cue processing. They also found that in individuals with high posterior alpha1 connectivity during rest, accuracy was influenced by this connectivity during final-word presentation. The topic should be of interest to speech researchers and cognitive and systems neuroscientists. The methods are robust. The manuscript is largely well written and easy to follow.”

We thank the reviewer for the positive feedback.

“The authors have adopted an interesting strategy with this manuscript, because many methodological details and statistics can only be found in the Supplemental Information. This leads to a very straightforward manuscript, but also bears the risk of burying important info in SI. I would suggest a more "traditional" approach and move important info into the main manuscript (cleaning of data, source localization, statistical main effects, even if not significant). It seems a very narrow picture to report only the most interesting results (the significant interactions) and not mention the other non-significant results.”

We followed the reviewer’s suggestion and moved the supplemental information which we deemed essential to the main text. We now describe *Data acquisition*, *Artefact rejection*, *EEG source and forward model construction*, and *Beamforming* within the Materials and Methods section. In addition, we added the non-significant main effects after reporting each brain-behavior interaction effect within the Results section. To assist the reviewer looking through the main text, these sections are all highlighted in yellow.

“Related to this, the discussion sometimes gives the impression that strong main effects were found, and not the actually found interactions, which indicate that there were connectivity-behavior links in a subset of participants. This should be expressed more carefully (and discussed) throughout to better reflect the statistical results.”

We agree with the reviewer. In the introductory section of the Discussion, we now clearly state that these effects were found in subsets of participants. In addition, we opened a new paragraph within the final section of Discussion to better reflect the statistical results, as it appears below:

“Importantly, our brain-behavior findings further the hitherto limited understanding of behavioral differences in how individuals cope with difficult listening situations. Nonetheless, these results should be interpreted carefully as they illustrate the behavioral relevance of cortical connectivity dynamics on a between-subjects trait-like level and in subsets of participants only, depending on individual’s resting-state baseline connectivity and its modulation during listening. Future studies will be important to investigate the state-dependency of cortical connectivity dynamics, in particular their trial-by-trial variability within individuals, to further pinpoint their link to listening behavior.”

“Other points:

1. How was the decision made to split the analysis into these specific frequency ranges? Please elaborate/justify.”

We apologize for being unspecific. This decision was based on prior knowledge on the spectral profile of cortical rhythms and their correlation structure.

Specifically, the study by Keitel and Gross (2016) provides a comprehensive understanding of the spectral modes of the human brain cortical rhythms. Their results suggest that cortical rhythms within ~7–30 Hz reflect different spectral modes with their dominant peaks at ~10 Hz, ~15 Hz, and ~20 Hz, that are characteristic to individual cortical areas and organize—according to their similarity—into large-scale networks (cf. alpha and beta line-graphs in Fig. 2 in Keitel and Gross, 2016, PLoS Biol).

In addition, previous studies on the correlation structure of cortical rhythms have consistently reported stronger power-envelope correlations within broad-band alpha (~7–14 Hz) and low-beta (~16–25 Hz) frequency ranges (e.g., Hipp et al. 2012, Nat Neurosci; Hipp and Siegel. 2015, Curr Biol; Siems et al. 2016, Neuroimage). In our data, the spectral profile of power-envelope correlations was in good agreement with these studies (Fig. 3; see below). This was further supported by the results of the between-subject reliability analysis as well as between-subject correlations with fMRI connectivity. Please note that, as suggested by Reviewer 2, we moved the spectral profile of whole-brain power-envelope correlations and their correlation with fMRI connectivity to the main text (Fig. 3; see below).

In the revised manuscript, we have elaborated on the decision made to split the analysis into these specific frequency ranges, as it appears bellow:

“It has been previously shown that cortical rhythms within ~7–30 Hz reflect different spectral modes with their dominant peaks at ~10 Hz, ~15 Hz, and ~20 Hz, that are characteristic to individual cortical areas and organize—according to their similarity—into large-scale networks (Keitel and Gross, 2016). In addition, previous studies on the correlation structure of cortical rhythms have consistently reported stronger power-envelope correlations within broad-band alpha (~7–14 Hz) and low-beta (~16–25 Hz) frequency ranges (e.g., Hipp et al. 2012; Hipp and Siegel. 2015; Siems et al., 2016). Accordingly, we focused our main analysis on three frequency bands within 7–24 Hz, namely $\alpha_{1}$(7–11 Hz), $\alpha_{2}$(11–14 Hz), and $\beta_{1}$(16–24 Hz).”

**Fig 3.** **Spectral profile of source EEG power-envelope correlations and their relationship with fMRI connectivity**. **(A)** Spectral profile of whole-brain mean connectivity during rest and listening task. At each frequency and per individual, mean connectivity was estimated as the upper-diagonal average of power-envelope correlation matrix thresholded at 10% of network density. Each line graph represents mean $\pm$ SEM of mean connectivity across all N = 154 participants. **(B)** Thirty participants have had performed the same task in fMRI [5]. Across these participants we tested the correlation between whole-brain mean connectivity derived from brain hemodynamics (i.e., mean Pearson correlation between fMRI band-passed filtered [0.06-0.12 Hz] BOLD signals) and whole-brain mean connectivity derived from EEG oscillatory source activity (i.e., mean power-envelope correlation at frequencies ranging from 2-32 Hz). Power-envelope correlations between EEG oscillatory sources show strongest positive correlation with fMRI connectivity within alpha and low-beta frequency range (grey frequency intervals).

“2. I am not quite sure what the message in the paragraph starting in line 67 is (initial submission). Is it that alpha and beta oscillations are modulated by slow time scales <.1 Hz? Could the authors clarify?”

We apologize for being unclear. The main message in this paragraph is that the envelope of ongoing alpha/beta oscillations in M/EEG shows similar dynamics to those observed in low-frequency BOLD activity in fMRI. These similarities can be summarized in the following three aspects: (1) During resting state these signals fluctuate mainly at frequencies blow 0.1 Hz (see, e.g., Fig.2e in Hipp et al., 2012, Nat Neurosci), (2) Their co-fluctuations (i.e., functional connectivity) correlate across M/EEG and fMRI, and (3) Their putative function seems to be coordination of activity between distributed cortical regions within a network during resting state and sensorimotor or cognitive tasks. To clarify, we revised this paragraph within the Introduction as it appears below:

“Nevertheless, we do not know yet whether and how attentive listening relies on coupling between neural oscillations across distributed cortical nodes. We here address this question within the unifying framework of intrinsic coupling modes by focusing on large-scale neural interactions that arise from coherent aperiodic fluctuations in signal envelopes that unfold at slow time scales (<0.1 Hz) (Engel et al., 2013). Specifically, in M/EEG recordings envelope fluctuations of ongoing alpha- and beta-band oscillations (~13–30 Hz) exhibit these dynamics, and their intrinsic co-fluctuations over time correlate with co-variations in brain hemodynamic activity during resting state (Mantini et al., 2007; Brookes et al., 2011; Hipp et al., 2012; Hipp et al., 2015). Accordingly, these neural dynamics have been proposed as one network mechanism whereby distant cortical regions functionally coordinate their activity to support performance in sensorimotor or cognitive tasks (Palva and Palva, 2012; Siegel et al., 2012).”

“3. Please briefly explain the use of "modular organization" (l. 183) in this context.”

Modular organization of a large-scale network refers to its decomposability into smaller subnetworks. The notion stems from contemporary graph theory, and it has been extensively applied to fMRI connectivity data in recent years (see Sporns and Betzel, 2016, Annu Rev Psychol, for review). These studies have shown that the topology of human “brain graphs” is not random, and that these indeed display a modular organization. Specifically, brain regions organize into clusters that are relatively densely *intra*-connected but sparsely *inter*-connected. This topological organization is thought to represent functional segregation across the human cortex (Sporns, 2013, Curr Opin Neurobiol). In our previous fMRI study, we found that modularity of brain networks was closely linked with individuals’ behavioral success in the same listening task as here.

To what degree the correlation structures of alpha/beta neural oscillations in electrophysiology show similar organization is not known. We thus investigated this in our data. We found that neither alpha nor low-beta connectivity displayed modular organization (S3 Fig; this is discussed in detail in the final section of Discussion). As suggested by the reviewer, we revised the respective paragraph in the Results section and explain the use of modular organization, as it appears below:

“We next investigated the modular topology of these correlation structures. Modularity of a large-scale network refers to its decomposability into smaller subnetworks. The notion stems from contemporary graph theory, and it has been extensively applied to fMRI connectivity data in recent years. Specifically, it has been shown that brain regions organize into clusters that are relatively densely *intra*-connected but sparsely *inter*-connected (Sporns and Betzel, 2016). This topological organization is thought to represent functional segregation across the human cortex (Sporns, 2013). The modular structure of a network can be estimated by the Newman optimization algorithm using the so-called modularity index (Blondel et al. 2008; Rubinov and Sporns, 2010): The more modular the network, the closer the index to 1. To what degree the correlation structures of alpha or low-beta neural oscillations show similar organization is not known.”

“4. The control analyses are great, but for the cross-validation results (starting line 306), I wonder what it means if 3 (and 1, respectively) out of 5 model iterations are significant (log BF larger than 1)? This does not seem to be the most robust? Could the authors add (a little) more information on how to reasonably interpret this?”

We agree with the reviewer in that the cross-validation results need more clarification. This analysis addresses two different yet complementary statistical questions:

1) Are the significant interaction effects reproducible when multiple random folds of data are used? Answering this question assures that the effects are not by chance, i.e., the model being fitted too closely to the peculiarities of the full sample. We examined this based on the model parameter estimate and its 95% confidence interval obtained from five model iterations. For the beta-band effect, consistent with the result obtained from the full sample, all five β estimates were significantly larger than zero (Fig. S10A, see below). For alpha-band effect, all five odds-ratios were smaller than one, with four estimates being significant (Fig. S10B). These results document within-sample reproducibility and robustness of the effects.
2) Addressing the first question does not take the number of observations and model complexity into account. Still, it could be that the model has a relatively high goodness-of-fit as a too complex model is fitted to the data. We thus further examined in how far this is the case by quantifying the relative strength of evidence (rather than statistical significance) in support of the alternative hypothesis (i.e., model with the interaction term) as compared to the null hypothesis (i.e., model without the interaction term). This was done by calculating the BIC approximation of the Bayes factor, i.e., exp([BIC(H0)–BIC(H1)]/2). In this calculation BIC imposes penalty on each model log-likelihood based on both number of observations and parameters. Following Harrold Jeffery’s scale for interpretation of BF (cf. Table 3 in Wagenmakers, 2007, Psychonomic Bulletin & Review), 0 < log-BF < 1 suggests weak evidence for H1, 1 < log-BF < 3 is positive, and 3 < log-BF < 5 is strong. Thus, our results overall support the more complex model that includes the interaction term (Fig. S10).

As suggested by the reviewer, within the respective paragraph of the Results we now briefly elaborate on the aim of this analysis and the overall interpretation of the results (these are highlighted in yellow). In addition, we improved data visualization in Fig. S10 to help interpretation of the results (see below) and added more details to its caption.

**S10 Fig Within-sample reproducibility and likelihood assessment of the brain-behavior interaction effects.** The reliability and robustness of the brain-behavior interaction effects (main text, Fig. 6) were investigated by randomly splitting the data into k = 5 non-overlapping folds. The strength and uncertainty (i.e., confidence interval) of the model’s parameter estimate was then examined when each fold of data was used. Since this analysis does not take the number of observations and model complexity into account, it could be that the model has a relatively high goodness-of-fir as a too complex model is fitted to the data. We thus examined the relative strength of evidence (rather than statistical significance) in support of the alternative hypothesis (i.e., model with the interaction term) as compared to the null hypothesis (i.e., model without the interaction term). This was done by calculating the BIC approximation of the Bayes factor, i.e., exp([BIC(H0)–BIC(H1)]/2). In this calculation BIC imposes penalty on each model log-likelihood based on both number of observations and parameters. Following Harrold Jeffery’s scale for interpretation of BF, 0 < log-BF < 1 suggests weak evidence for H1, 1 < log-BF < 3 is positive, and 3 < log-BF < 5 is strong [104]. *β*: Slope parameter estimates from linear mixed-effects model. *OR*: Odds ratio parameter estimates from generalized linear mixed-effects models.

“5. Many of the supplemental figures miss detailed captions. Could the authors please add these?”

We apologize for incompleteness. Detailed captions are now provided for all supplemental figures. These changes are highlighted in yellow.

“6. Please explicitly report the final sample size and demographic description of the final sample.

7. Related to this, I would suggest detailing the actual sample size in the abstract, instead of the initial number of participants before exclusion.”

We apologize for being unclear. We now explicitly report the final sample size and demographics in the Participants section, as it appears below:

“Reported results are from N = 154 participants (right-handed German native speakers, age range = 39–80 yrs, median age = 61 yrs, 62 males).”

The final sample size is also stated in the Abstract. These changes are highlighted in yellow.

**Reviewer 2**

“The paper complements an earlier fMRI connectivity study by the same authors in which they used resting state data and an attentional listening paradigm. The group now provides an analysis of amplitude envelope coupling in EEG data using the same task paradigm. While the EEG recordings use the minimum number of channels (64 electrodes) required for meaningful source analysis, an interesting feature of the study is a large sample, since data from more than 150 subjects were included. The results replicate known resting state envelope coupling patterns. Furthermore, the study shows that auditory attention modulates envelope coupling in the beta band, leading to higher functional connectivity of frontoparietal regions compared to the resting state. For subgroups of the subjects, connectivity values predict performance in the task.”

We thank the reviewer for positively evaluating our work, and we hope to be able to convince the reviewer further of the added value from these findings.

“The state-of-the-art is not properly covered in the introduction; for instance, there is substantial work on network dynamics during crossmodal attentional shifts involving the auditory system which are not covered here.”

We agree with the reviewer in that, knowing the broad readership of the journal, it is important to go beyond the auditory (and visual) attention literature and cover the state-of-the-art on network dynamics of cross-modal attention too. In the light of the reviewer’s comment, we found the studies by Hipp and colleagues (2011, Neuron) as well as Fries and colleagues (2016, Sci Rep) most relevant, as they provide evidence for the role of coherence between neural oscillations in cross-modal (i.e., audio-visual) perception and attention. We now briefly cover this topic within the Introduction, as it appears below (please note that, for the convenience of the reviewer, these changes are all highlighted in green within the main text):

“[…] Accordingly, these neural dynamics have been proposed as one network mechanism whereby distant cortical regions functionally coordinate their activity to support performance in sensorimotor or cognitive tasks (Palva and Palva, 2012; Siegel et al., 2012). In line with this proposal, coherent neural activity during task states has been shown to subserve multisensory perception, attention, and cognitive control (Hipp et al. 2011, Fries et al., 2016). How intrinsic neural oscillations regulate their envelope coupling to support behavioral adaptation to attentive listening is unknown.”

“Also, the statement that studies on visuo-spatial attention have provided „initial evidence" on a role of coupling for attention (p.6, line 65) is misleading; there is substantial evidence, and in my view it would rather be fair to say that these studies have pretty much resolved the role of coupling for attentional selection.”

We agree that the term “initial evidence” could be misleading. We thus revised this paragraph within the Introduction, as it appears bellow:

“Neurophysiological studies on visuo-spatial attention provide substantial guiding evidence on how functionally specialized coupling between distant cortical nodes involving prefrontal regions underlie the dynamics of attentional selection and control (Popov et al., 2017, J Neurosci; Marshall et al., 2015, PLoS Biol; Bonnefond et al., 2017, eNeuro; see Fiebelkorn and Kastner, 2020, Annu Rev Psychol, for review). Nevertheless, we do not know yet whether and how attentive listening relies on coupling between neural oscillations across distributed cortical nodes.”

“In the task design, spatial attention and feature-based attention are mixed; the rationale for combining these two aspects is not clearly presented.”

The spatial-attention cue was designed to experimentally invoke selective or divided auditory attention, with the selective condition having two levels: left or right. We thus presented the spatial-attention cue in the from of a circle equally segmented into two lateral halves and used black color for either half on selective trials (i.e., “attend left” or “attend right”) or grey for both sides on divided trials (supplementary text: Dichotic listening task). During the experiment, the color feature of the spatial-attention cue was not behaviorally relevant to the main task, namely reporting the final word of one of the dichotically presented sentences (i.e., left/right). In fact, on selective trials only the side of the black half was 100% informative.

Indeed, our analyses on the neural level strongly support that the spatial-attention cue has invoked auditory selective attention as expected. This was in fact one of the main questions asked in our large-scale study which is thoroughly addressed in Tune et al., 2021 (Nat Commun, in press) and is also covered within the scope of the present study (main text: Control analysis; Fig. S7). In brief, we investigated alpha power dynamics using attentional modulation index (AMI) defined as AMI = (α-power_attendL_ $-$ α-power_attendR_) / (α-power_attendL_ $+$ α-power_attendR_). During presentation of the spatial-attention cue, this analysis revealed the strongest hemispheric lateralization of alpha power within the auditory cortex (see below).

“The age range of subjects included is pretty broad; it would be interesting to know whether there were significant age-related differences for resting state or task-related envelope coupling.”

This is indeed an interesting question. We directly tested the Spearman’s correlation between age and mean resting state or task connectivity derived from each frequency band. As illustrated below, none of these correlations were significant. We report these correlations within the Results section, as it appears below:

“As mentioned earlier, in our brain-behavior models we controlled for participants’ chronological age and hearing thresholds. In additional analyses we explored the possible correlations between either of these two covariates with mean resting state or task connectivity per frequency band. None of these correlations were significant ($\alpha_{1,rest}$: Spearman’s ρ = 0.01, p = 0.89; $\alpha_{1,task}$: ρ = $-$0.01, p = 0.99; $\beta_{1,rest}$: ρ = 0.02, p = 0.8, $\beta_{1,task}$: ρ = $-$0.1, p = 0.2).”

“Overall, the study is solid and uses an interesting paradigm. However, on the background of previous work on functional connectivity and attention, novelty is limited.”

We thank the reviewer for the positive feedback on our study and paradigm. With respect to the novel aspects of the study, we would like to draw the reviewers’ attention to the following points.

The present study treats attentive listening in a dual-talker situation as an adaptive behavior (Alavash, Tune, Obleser, 2019, PNAS). Our guiding hypothesis is that, for the individual listener to adapt successfully to a listening challenge, intrinsic brain networks need to adapt accordingly. In the light of the previously proposed role of large-scale neural interactions in adaptive behaviors (Palva and Palva, 2012, TICS; Siegel et al., 2012, Nat Rev Neurosci; Engel et al., 2013, Neuron), we predicted that large-scale envelope correlations between neural oscillations within alpha-beta frequency range change relative to its intrinsic resting-state baseline to support attentive listening behavior. To our knowledge, empirical evidence in support of these large-scale neural coupling dynamics and their functional relevance to adaptive behavior has not been provided yet.

To test our hypothesis, we took a comprehensive approach to investigate the spectral, spatial, and temporal dynamics of the envelope correlations (i.e., their momentary changes during attentive listening relative to rest). Accordingly, our study also offers a novel yet feasible network identification approach which can be used to investigate large-scale neural dynamics that underlie different adaptive behaviors. In this way, we believe that our work suits well the broad readership of the journal.

Reassuringly, our approach allowed us to:

1) Functionally specify alpha and beta connectivity dynamics during attentive listening in terms of momentary task states (i.e., attention to target speech vs. anticipation of the upcoming target speech, respectively) and their large-scale cortical network configuration (i.e., posterior vs. frontoparietal, respectively).
2) Provide new insights into how these functionally specific connectivity dynamics link to the behavior of a group of listeners.

Collectively, these points add to the current knowledge on functional connectivity and attention by providing evidence in support of two distinct yet complementary large-scale networks of intrinsic neural oscillations that underlie attentive listening behavior.

To further highlight the novel aspects of our study, we added the following paragraph within the final section of the Discussion:

“Our key finding is that connectivity between frontoparietal low-beta and posterior alpha oscillations relative to their resting-state baseline allow predicting distinct aspects of individuals’ listening behavior. To our knowledge, empirical evidence in support of these neural coupling dynamics and their functional relevance to goal-directed behavior has not been provided yet. Thus, both studies share a common thread: dynamics of large-scale cortical networks retain the information that could predict trait-like individual differences in attentive listening. In this way, our study also offers a novel yet feasible network identification approach which can be used to investigate where, when, and how large-scale neural dynamics underlie goal-directed behaviors.”

Finally, as it has been discussed in our initial submission (see below), we think that the present study also has novel aspects particularly important for the field of auditory neuroscience:

“Attentional modulation of neural responses within auditory cortex has been extensively investigated along different lines of research in auditory neuroscience (cf. Tune et al., 2021, for a study on auditory alpha power and neural tracking of speech using the same data and cohort as here). Nevertheless, large-scale network dynamics of the listening brain has remained underexplored, particularly on the neurophysiological level. This is mainly due to the methodological challenges inherent to connectivity analysis (Schoffelen and Gross, 2009; O'Neill et al., 2015; Palva et al., 2018). Here we carefully took these considerations into account. This eventually allowed us to map and functionally characterize large-scale neural connectivity within alpha and low-beta frequency channels whereby distant cortical nodes tuned into attentive listening according to the listener’s goal. Importantly, our brain-behavior findings further the hitherto limited understanding of behavioral differences with which individuals cope with difficult listening situations.”

**Reviewer 3**

“This study investigated oscillatory power correlations in elderly adults using EEG. The study found that frontoparietal beta-band connectivity was increased while posterior alpha-band connectivity was decreased during challenging dual-talker listening task. The manuscript is well written and coherent, the results being presented in a clear manner. The analyses seem also well performed and there is extensive amount of control analysis included which I found very convincing. The illustration of the results could be improved to better convey the message but overall the study is of very good quality.”

We thank the reviewer for the supportive feedback.

“Major
In the abstract it is written that "connectivity between intrinsic neural oscillations extracted from source-reconstructed electroencephalography is top-down regulated during a challenging dual-talker listening task." However, there is no directional analysis in this study and the sentence should be reformulated to reflect this. The localization of the connectivity is suggestive of the top-down influence, but this should be separately tested.”

We agree with the reviewer. We thus re-phrased this sentence, as it appears below:

“[…] connectivity between intrinsic neural oscillations extracted from source-reconstructed electroencephalography is regulated according to the listener’s goal during a challenging dual-talker task”.

We also made two additional similar changes within the final section of the Discussion. To assist the reviewer looking through the main text, these changes are highlighted in cyan.

“There is no summary of the connectivity results, which would show connectivity changes as a function of frequency for different conditions e.g. as time-frequency representations of connectivity. Thus, it is not evident that the effects would be found in the alpha and beta bands now used in the analyses. These data might be presented in Fig S2d-e but there is no legend for these, so it is unclear. I suggest that these are moved to main figures, if they are the main data of the findings.”

We thank the reviewer for this suggestion. We moved Fig S2d-e to the main text, i.e., the first section of the Results where we report on the spectral profile of connectivity. Additionally, we provide detailed caption for this figure (see below). Please note that, as also pointed by the first reviewer, we added detailed captions to all supplementary figures. We apologize for incompleteness.

**Fig 3.** **Spectral profile of source EEG power-envelope correlations and their relationship with fMRI connectivity**. **(A)** Spectral profile of whole-brain mean connectivity during rest and listening task. At each frequency and per individual, mean connectivity was estimated as the upper-diagonal average of power-envelope correlation matrix thresholded at 10% of network density. Each line graph represents mean $\pm$ SEM of mean connectivity across all N = 154 participants. **(B)** Thirty participants have had performed the same task in fMRI [5]. Across these participants we tested the correlation between whole-brain mean connectivity derived from brain hemodynamics (i.e., mean Pearson correlation between fMRI band-passed filtered [0.06-0.12 Hz] BOLD signals) and whole-brain mean connectivity derived from EEG oscillatory source activity (i.e., mean power-envelope correlation at frequencies ranging from 2-32 Hz). Power-envelope correlations between EEG oscillatory sources show strongest positive correlation with fMRI connectivity within alpha and low-beta frequency range (grey frequency intervals).

“The lack of low-frequency oscillations to the final world could also arise if the final world is not time-locked to the beginning of the sentence and thus the oscillatory signals being averaged out. Please clarify how the timing of the very long trials with variable position of the final words were ensured?”

We apologize for being unclear. In the dichotic listening task, each sentence pair was temporally aligned by the onset of the two task-related sentence-final words which led to slightly asynchronous sentence onsets. The histogram of the final word onsets is plotted in Fig 1 (see below). This is now clarified within the Materials and Methods section.

We would also like to draw the reviewer’s attention to our in-depth analysis of auditory low-frequency (< 8 Hz) responses during sentence presentation (Tune et al., 2021, Nat Commun, in press). In brief, in the same data and using temporal response functions, we were able to show that neural tracking of the speech envelope was modulated by attention. Related to the reviewer’s comment above, this modulation became increasingly positive during the second half of sentence presentation with its highest peak around final word onset (Fig 4c in Tune et al., 2021). Thus, when analyzing low-frequency neural activity, there is indeed evidence for auditory responses to the final word despite the slight asynchronous sentence presentation.

In the present study, however, we analyzed correlations between power-envelopes of neural responses. This measure is not sensitive to the timing of neural responses, but rather to co-fluctuations in their sluggish power envelopes (after removing their zero-lag correlations). Therefore, the lack of low-frequency connectivity is arguably due to the characteristic of the measure used rather than temporally slightly variable sentence presentation.

“Connectivity data is presented as matrix correlation coefficients between different lobes as well as nodal connectivity overlaid in the brain surface. I find these figures suboptimal. The connectivity estimates presented in Figs 4, 5 are not informative of the anatomy nor strength of the connections, because the parcels are not identifiable neither from the matrixes (which should have the labels at least in some panels) nor from the brain which are really blurred and not revealing the brain areas. Maybe the authors could add some land-mark brain regions relevant to auditory attention and semantic processing. Also color scale could be more informative to reveal differences in the strength of the connectivity.”

We agree with the reviewer in that parcels are not identifiable according to their neuroanatomy. We thus followed the reviewer’s suggestion and added landmarks to one of the brain surfaces as a template. These include superior temporal sulcus (STS), perisylvian language area (PSL), inferior parietal lobule (IPL), frontal eye field (FEF), dorsolateral prefrontal cortex (dlPFC), and inferior frontal gyrus (IFG) (see below).

Please note that the strength of connectivity can be directly inferred from S4 Fig. To better illustrate connections that show modulation in their connectivity strength, we now additionally visualize the connectograms in a new supplemental figure S11 Fig. Please see the response to the next comment for details.

“Nodal connectivity is now used to plot the cortical regions with connectivity modulations. Strength based metrics are not the best to use as they are influenced by the source-leakage (the poor sources have the largest signal). Centrality measures are more robust in this respect and they also give some insight of the network structure and its relevance to information processing. I would thus suggest trying alternative approaches to visual the data to increase the clarity of the results.”

We agree with the reviewer in that investigating how measures of centrality are modulated could provide additional insights into the current findings. We thus followed the reviewer’s suggestion and revisited our data.

Knowing the statistical nature of connections in our case (i.e., power-envelope correlations), we focused on two nodal measures of centrality, namely degree centrality and eigenvector centrality, and refrained from using measures defined based on paths or distances such as betweenness centrality (such measures are more appropriate for studying structural connectivity, cf. Rubinov and Sporns, 2010, Neuroimage). Accordingly, we investigated whether degree or eigenvector centrality are modulated during processing of the spatial cue or final word relative to resting state.

The new supplemental figure S11 summarizes the results. In brief, the spatial pattern and direction of the modulations were highly similar across the two centrality measures. However, these modulations were weaker as compared to the modulations previously found for nodal connectivity strength (cf. Fig 5, main text) and did not survive correction for multiple comparisons across nodes. We note that, in our main results, modulations of connectivity strength are found also across distant cortical nodes, i.e., frontoparietal connections (beta-band effect) as well as occipitotemporal or between-hemispheric connections (alpha-band effect; S11 Fig A, black connections, see below). Thus, it is less likely that these modulations are influenced by source leakage.

Taken together, these results indicate that the topology of power-envelope correlations were not significantly altered during the listening task relative to rest. This reiterates our previous observation that, the topology of these correlations did not display modular segregation which would otherwise suggest centrality at the level of subnetworks or modules (e.g., nodes having relatively high within-module z-score). Accordingly, our data suggest that power-envelope correlations within alpha and low-beta frequency range have an intrinsic structure whose strength (but not topology) is modulated by task context. We mention to these new results within the Discussion, as it appears below:

“We additionally investigated whether measures of network centrality, namely degree centrality and eigenvector centrality, were modulated during processing of the spatial cue or final word relative to resting state. In contrast to nodal connectivity, neither of these topological measures showed significant modulations during the listening task (S11 Fig). Taken together, our data suggest that the source-EEG power-envelope correlations within alpha and low-beta frequency range have an intrinsic structure whose connectivity strength (but not topology) is modulated by task context.”

**S11 Fig. Network topology and centrality of α/β oscillations under rest and attentive listening.** **(A)** Network topology per frequency band and condition. Each connectogram corresponds to its connectivity matrix counterpart and is obtained by setting the connection weights to 1 for correlations higher than 95-th percentile, and to 0 otherwise (i.e., a sparse binary graph thresholded at 5% of density). Nodes correspond to cortical parcels as in [35] and are grouped according to their cortical lobes. The frontoparietal and posterior cortical nodes showing significant modulation of $\beta_{1}$ and $\alpha_{1}$ connectivity strength, respectively (main text, Fig. 5), are depicted in black. **(B)** Comparison of nodal degree centrality between each task interval and rest using permutation tests. Degree centrality is defined as each node’s number of connections (also referred to as node’s degree). This measure quantifies relative importance of nodes: nodes having large number of connections, hence high degree centrality, can be considered as central coordinators in the functional network. In contrast to nodal connectivity strength, nodal degree was not significantly modulated when tested across all cortical nodes (brain surfaces; significance level: p_FDR_<0.01). **(C)** Comparison of eigenvector centrality. This metric is more specific than degree centrality as it also takes the relative importance of a node’s neighbors into account. Eigenvector centrality of node $i$ is equivalent to the $i$th element in the eigenvector corresponding to the largest eigenvalue of the adjacency matrix. Nodes having high eigenvector centrality are high-degree nodes whose neighbors also have relatively large number of connections. Eigenvector centrality was not significantly modulated when tested across all cortical nodes. *LH*: left hemisphere, *RH*: right hemisphere, *STS*: superior temporal sulcus, *PSL*: perisylvian language area, *IPL*: inferior parietal lobule, *FEF*: frontal eye field, *dlPFC*: dorsolateral prefrontal cortex, *IFG*: inferior frontal gyrus.

“Minor
Morlet wavelets from 1-32 Hz were used to analyse oscillation power. Fig S7 clearly shows that there would be oscillations also above the 32Hz so why this cut-off was used? Gamma-band oscillations from 30-100 Hz are known to be related to the processing of auditory semantic information.”

The focus of the present study is on large-scale network interactions which are assumed to support participation of relevant cortical regions in a task (in our case, adaptation to attentive listening). Network interactions as such have been previously theorized to emerge from co-fluctuations in ongoing neural or hemodynamic activities that unfold at slow time scales (<0.1 Hz; Engel et al., 2013, Neuron). In M/EEG recordings, these dynamics have been found to be dominated by envelope fluctuations of alpha- and beta-band oscillations (~13–30 Hz) (main text, Introduction). Indeed, in our data we observed that power-envelope correlations were strongest within alpha and low-beta frequency range and appeared weaker for frequencies above 24 Hz (main text, Fig 3A).

This relative decrease in power-envelope correlations in higher frequencies has been observed in several other studies and is, to some extent, due to high susceptibility of EEG to muscle artefacts which is detrimental to estimating gamma-band connectivity sensibly (see Siems et al., 2016, Neuroimage, for a detailed discussion on this topic). In addition, gamma-band activity is generally associated with local neural computations that manifest in sensory or motor cortices (see, e.g., Koppel et al., 2000, PNAS; Bastos et al., 2015, Neuron). Since M/EEG recordings average activity over large neural populations, they have low sensitivity to spectral components with a focal spatial coherence (Hipp et al., 2012, Nat Neurosci).

In our experiment, it could well be that gamma activity increases in auditory and semantic cortical areas. However, measuring the correlation structure of these (focal) activities using EEG, if possible, would require a careful consideration of muscle artefacts (Hipp and Siegel, 2013, Front Hum Neurosci).
