# Supplementary material for: Dynamic large-scale connectivity of intrinsic cortical oscillations supports adaptive listening in challenging conditions
Source: PLoS Biol. 2021 Oct 11;19(10):e3001410. doi: 10.1371/journal.pbio.3001410 (PMC8530332; doi:10.1371/journal.pbio.3001410)
Supplement: Attachment — Submitted filename: Response_to_reviews_R2.docx [file pbio.3001410.s019.docx]

Response letter to the reviewers’ comments on “Dynamic large-scale connectivity of intrinsic cortical oscillations supports adaptive listening in challenging conditions” submitted to *PLOS Biology* in March 2021.

Manuscript No.: PBIOLOGY-D-21-00518R1

Authors: Mohsen Alavash*, Sarah Tune, Jonas Obleser*

*Corresponding author: Department of Psychology, University of Lübeck, 23562 Lübeck, Germany

Revised manuscript submitted to journal in August 2021 (Revision 2).

**Reviewer 1**

“The authors responded well to the comments and incorporated changes to make analyses and results even clearer. The answer to 'why these frequency bands', is not very specific, and does not explain why e.g. alpha was split into a higher and a lower band (although I think there is precedence and reason to do this, but this is not explained/justified in the manuscript).”

We now specifically explain that splitting alpha into lower and higher bounds is based on its spectral variation (i.e., alpha peak frequency) across cortex, as it appears below:

“This frequency range overlaps with the broad-band alpha (7–14 Hz) and the lower end of beta band (16–24 Hz). The dominant frequency of alpha-band oscillatory activity appears to vary across cortex and task conditions (Haegens et al., 2014; Keitel and Gross, 2016). Specifically, while alpha oscillations at the lower end of the frequency range (7–11 Hz) have been recorded from occipitoparietal sources during rest or visual tasks and temporal sources during auditory tasks, these oscillations appear slightly faster over sensorimotor cortices (Haegens et al., 2011; Obleser & Weisz, 2012; Clayton et al., 2018). Accordingly, we focused our main analysis on three frequency bands within 7–24 Hz, namely $\alpha_{1}$(7–11 Hz), $\alpha_{2}$(11–14 Hz), and $\beta_{1}$(16–24 Hz).”

“Also, there is one point that needs clarification, which is the reference to the Keitel & Gross 2016 study. This previous study found spectral modes between 1 and 120 Hz (most of which were in the alpha band), not "~7-30 Hz" as stated in the manuscript/reply. This should be amended.”

We agree that our previous citation could misrepresent the actual work by Keitel & Gross, 2016. In the current version, we revised our citation so that it refers to the main finding rather than the details of the spectral analysis, as it appears below:

“Spectral modes as such have been shown to be characteristic to individual cortical areas and organize—according to their similarity—into large-scale networks (Keitel and Gross, 2016).”

“I was also asked to assess the responses to concerns by Reviewer 2. Regarding their first point ("state-of-the-art", e.g. "network dynamics during crossmodal attentional shifts"), the authors now provide background on a few previous findings, although it is difficult to say whether these are what Reviewer 2 had in mind.”

Thank you for investing extra time on this review. We were also not sure which studies the reviewer had in mind. As mentioned in our previous response, we found these two studies the most relevant.

“Regarding the novelty aspect more broadly, the results probably do not seem surprising, because alpha and beta oscillations are commonly associated with attention and performance. However, the specific connectivity method (envelope correlations in EEG) in combination with the specific auditory behavioural paradigm yields a nice set of results.”

Thank you for your positive feedback. As we have already elaborated on the novel aspects of this study, and in agreement with you, we believe that our network identification approach—i.e., focusing on changes in envelope correlations of EEG oscillatory responses during listening relative to rest—and the functional relevance of these dynamics to individual listening behavior distinguishes this study from previous work.

“Reviewer 2 also asked to explain the rationale for combining spatial and feature-based attention in the task. The authors elaborate on the use of spatial attention but do not explain why they combined this with feature-based attention.”

Our experimental design does not offer any feature which could be combined with the spatial information or would be relevant to the task in any other way. We understood the comment by Reviewer 2 as a reflection on the color feature of the spatial cue. Our previous response precludes any possibility for this feature to be combined, if at all, with spatial information. In our 2-by-2 design, only spatial attention and semantic prediction were experimentally manipulated. None of these cues tap into feature-based attention. In addition, sentences used during dichotic auditory presentation were spoken by the same female talker, which also precludes the possibility that the participants have used non-spatial auditory features (such as pitch or gender of the talker) to guide their focus of attention. In the current version of the manuscript, we write this additional information in the second paragraph of the Results section. This is highlighted in yellow.

“The remaining points are answered satisfactorily, in my opinion.”

Thank you.
